# Dynamics of supracellular keratin bundling and nuclear uncaging in stretched epithelia

**DOI:** 10.1038/s41567-026-03371-8

**Published:** 2026-07-27

**Authors:** Tom Golde, Marco Pensalfini, Nimesh Chahare, Pere Roca-Cusachs, Gerhard Wiche, Guillaume T. Charras, Marino Arroyo, Xavier Trepat

**Affiliations:** 1 Institute for Bioengineering of Catalonia (IBEC), The Barcelona Institute of Science and Technology (BIST), Barcelona, Spain; 2 Laboratori de Càlcul Numeric (LaCàN), Universitat Politècnica de Catalunya-BarcelonaTech, Barcelona, Spain; 3 School of Engineering and Materials Science, Queen Mary University of London, London, UK; 4 Columbia University, New York, NY USA; 5 Facultat de Medicina, Universitat de Barcelona, Barcelona, Spain; 6 Department of Biochemistry and Cell Biology, Max Perutz Labs, University of Vienna, Vienna, Austria; 7 London Centre for Nanotechnology, University College London, London, UK; 8 Institute for the Physics of Living Systems, University College London, London, UK; 9 Department of Cell and Developmental Biology, University College London, London, UK; 10 Centre Internacional de Mètodes Numèrics en Enginyeria (CIMNE), Barcelona, Spain; 11 Institució Catalana de Recerca i Estudis Avançats (ICREA), Barcelona, Spain; 12 Centro de Investigación Biomédica en Red en Bioingeniería, Biomateriales y Nanomedicina (CIBER-BBN), Barcelona, Spain

**Keywords:** Biological physics, Biopolymers in vivo, Computational biophysics

## Abstract

There is broad consensus that intermediate filaments, such as keratin, play a key role in protecting cells and tissues from large deformations. However, little is known about how they fulfil this function. Here we show that epithelial cells slowly adapt to stretching through a coupling of a star-bundling transition of keratin filaments with the escape of the nucleus from its keratin cage. The bundling transition begins with a depletion of keratin filaments at tricellular junctions followed by a progressive accumulation in thick bundles that bisect cell–cell junctions. Bundling is a cooperative process that initiates in a few scattered cells and propagates to their neighbours, leading to the growth of multicellular clusters that contain a percolated network of thick keratin bundles. Bundling dynamics are slow and strongly influenced by the interaction between actin and keratin. Informed by a computational model, we provide evidence that keratin bundling generates a compressive stress on the nucleus, which is relaxed by nuclear escape from the keratin cage. The topological transitions identified here provide epithelia with a multiscale mechanism to adapt to sustained stretching.

## Abstract (web summary)

Keratin intermediate filaments form a cage around the nucleus to protect it from stress. Now it is shown that under large deformations the filaments bundle and ultimately release the nucleus from its cage.

## Main

Intermediate filaments, such as keratin and vimentin, constitute an essential class of cytoskeletal polymers widely expressed in animal cells. Owing to their hierarchical structure, individual filaments and their networks exhibit exceptional mechanical properties: they can stretch several times their original length but undergo pronounced stiffening at higher strains^1–5^. At the single cell level, intermediate filaments support cell integrity by resisting both tensile and compressive stresses and by shielding the nucleus from its mechanical microenvironment^6–12^. In epithelial tissues, keratin intermediate filament networks are responsible for strain-stiffening and are crucial for maintaining tissue cohesion during large deformations^13–17^. Collectively, these findings have led to the growing consensus that, although actin filaments largely determine cell and tissue mechanics at small deformations, intermediate filaments play a key role in governing cellular responses to larger strains^17–20^.

Although extensive functional and mechanical evidence now supports this consensus, the reorganization of intermediate filament networks in response to mechanical stretching and their specific role in providing mechanical protection remain poorly understood. In relaxed epithelial monolayers, keratin filaments form two interconnected networks, one lining the cell cortex and the other enveloping the nucleus. The cortical keratin network, commonly referred to as the rim, connects adjacent cells through desmosomes and is linked to the nuclear-enveloping network through short filament bundles known as spokes. This rim-and-spoke structure relies heavily on the crosslinking protein plectin, which bridges the actin and keratin cytoskeletons and couples keratin to the nucleus via nesprins^21,22^. Upon mechanical stretching, the organization of these networks is transformed^23–28^. In MDCK monolayers, cells exposed to large strains display long, thick keratin cables that withstand tension and form supracellular networks^19,29^. Long keratin cables spanning several cells have also been found in developing embryos, which indicates that they have a protective and stabilizing role^30^. How such keratin structures form under stretching, how they organize into supracellular networks and how they modulate the mechanical properties of the tissue are not known.

Here we combine experimental and theoretical approaches to study the dynamic reorganization of the keratin cytoskeleton and its coupling to the nucleus during sustained stretching of epithelial monolayers. Using a new device to stretch curved cell monolayers, we found that the keratin network adapts to prolonged mechanical loading by undergoing a slow bundling transition. The kinetics of this transition is largely governed by the actin–keratin interaction. Strikingly, we show that the bundling transition leads to the full escape of the nucleus from its keratin cage. A computational model explains this behaviour in terms of a compression-by-tension mechanism in the entangled keratin network. Finally, we demonstrate that the drastic structural remodelling of the keratin cytoskeleton is a collective supracellular process driven by a nucleation-and-growth mechanism. Our study unveils a new multiscale response of cellular monolayers to sustained stretching, wherein the nucleus is ultimately unshielded from the keratin cytoskeleton.

## Results

### Keratin undergoes the star-bundling transition in spontaneous domes

**Fig. 1:**
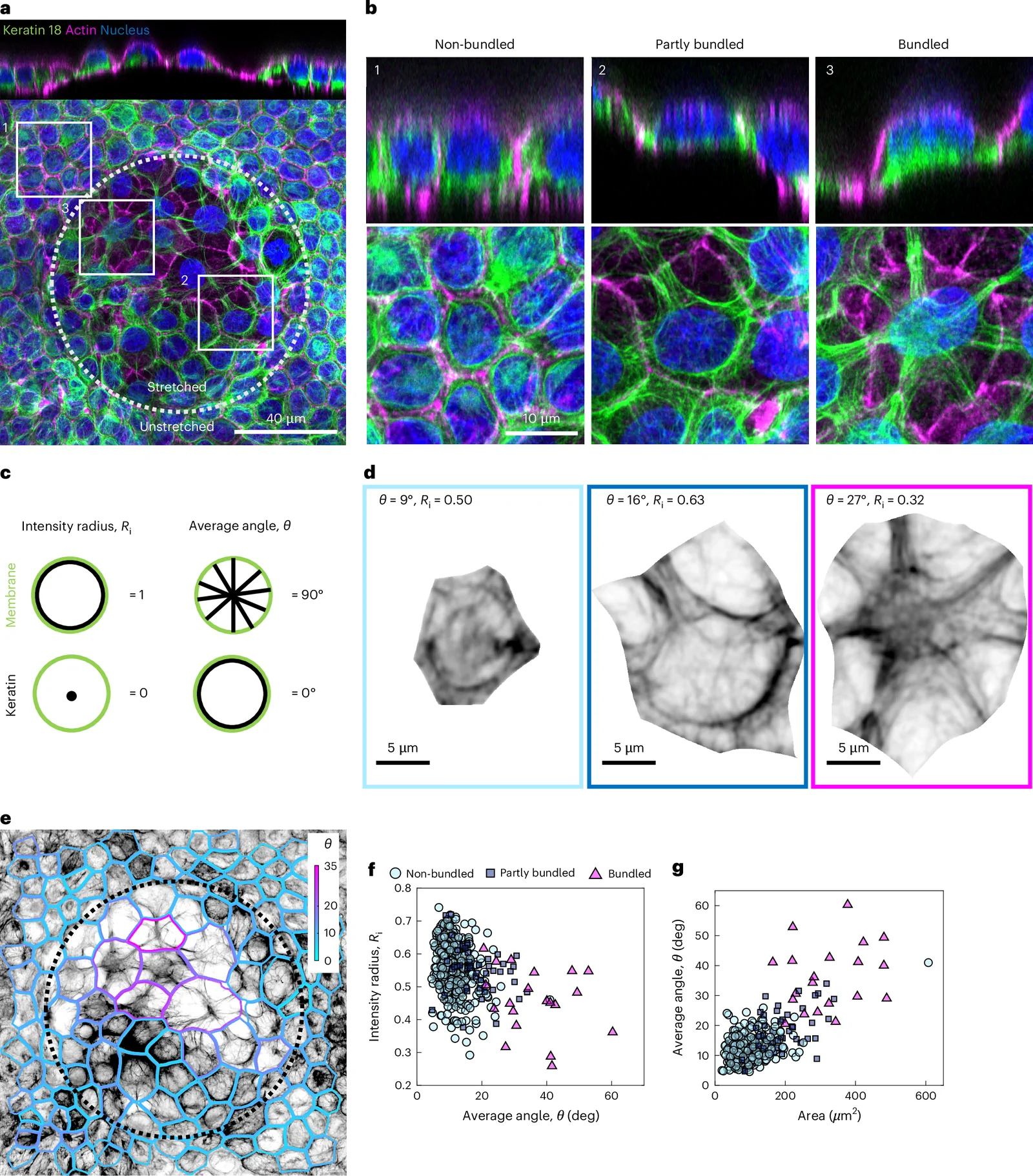
Keratin bundles in spontaneous domes. **a**, Spontaneous dome of MDCK cells expressing keratin-18–GFP (green) stained for F-actin (magenta) and nuclei (Hoechst, blue). Top: lateral view. Bottom: maximum intensity projection with dome footprint marked with a white dotted circle. **b**, Zoomed-in views of the three regions marked with white rectangles in **a**, showing non-bundled, partly bundled and bundled cells. Top: lateral views. Bottom: maximum intensity projections. **c**, Schematic illustration of the intensity radius *R*_i_ and average angle *θ*. **d**, Keratin-18–GFP images of the three cells shown in **b**. **e**, Keratin-18–GFP intensity in the spontaneous dome shown in **a**. The dome footprint is marked with a black dotted circle. The segmented cell boundaries are colour-coded according to the average angle *θ*. **f**, Intensity radius plotted against the average angle (*R* = −0.34, *P* = 2.9 × 10^−15^). **g**, Average angle *θ* plotted against cell area (*R* = 0.71, *P* = 6.3 × 10^−79^). *n* = 509 cells in **f** and **g**. *R* values are Pearson’s correlation coefficient. Source data

**Extended Data Fig. 1:**
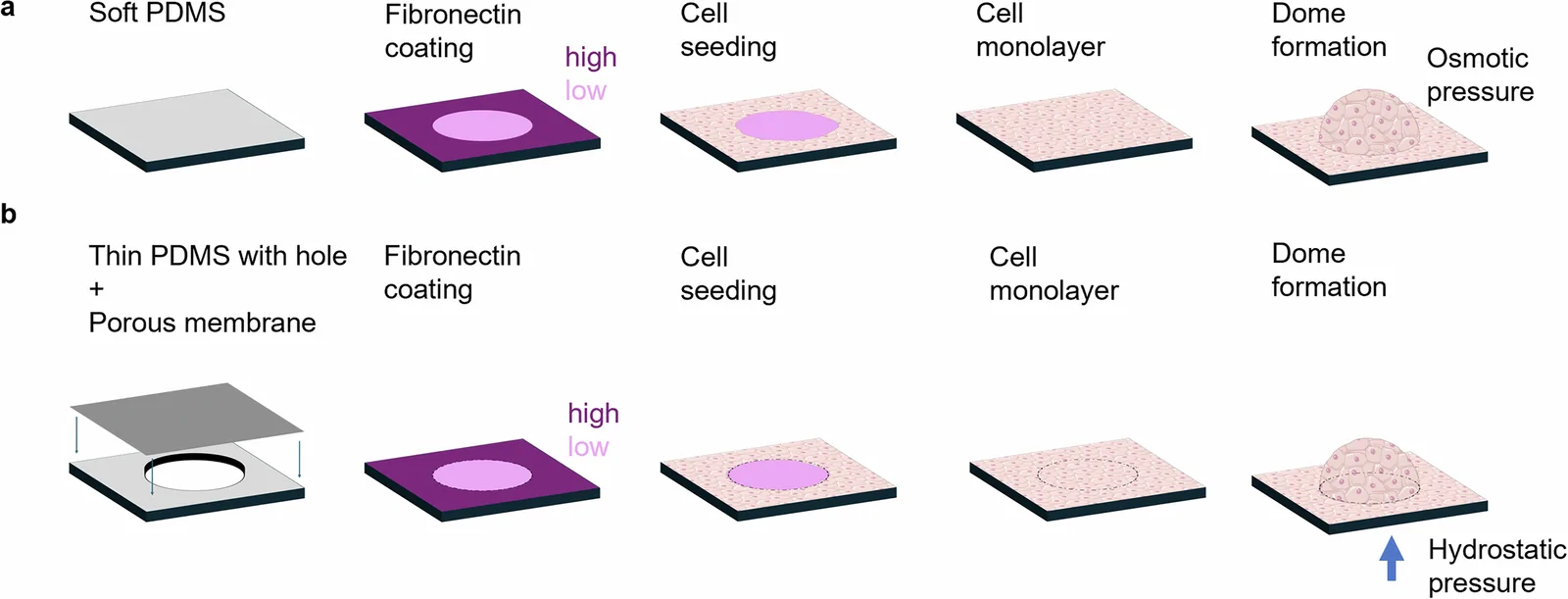
Experimental design of spontaneous and directed domes. **a**, Illustration of the different steps in preparing micropatterned spontaneous domes. **b**, Illustration of different steps in preparing micropatterned directed domes. See methods section for details. Credit: cell monolayer illustration, NIAID NIH BIOART.

**Extended Data Fig. 2:**
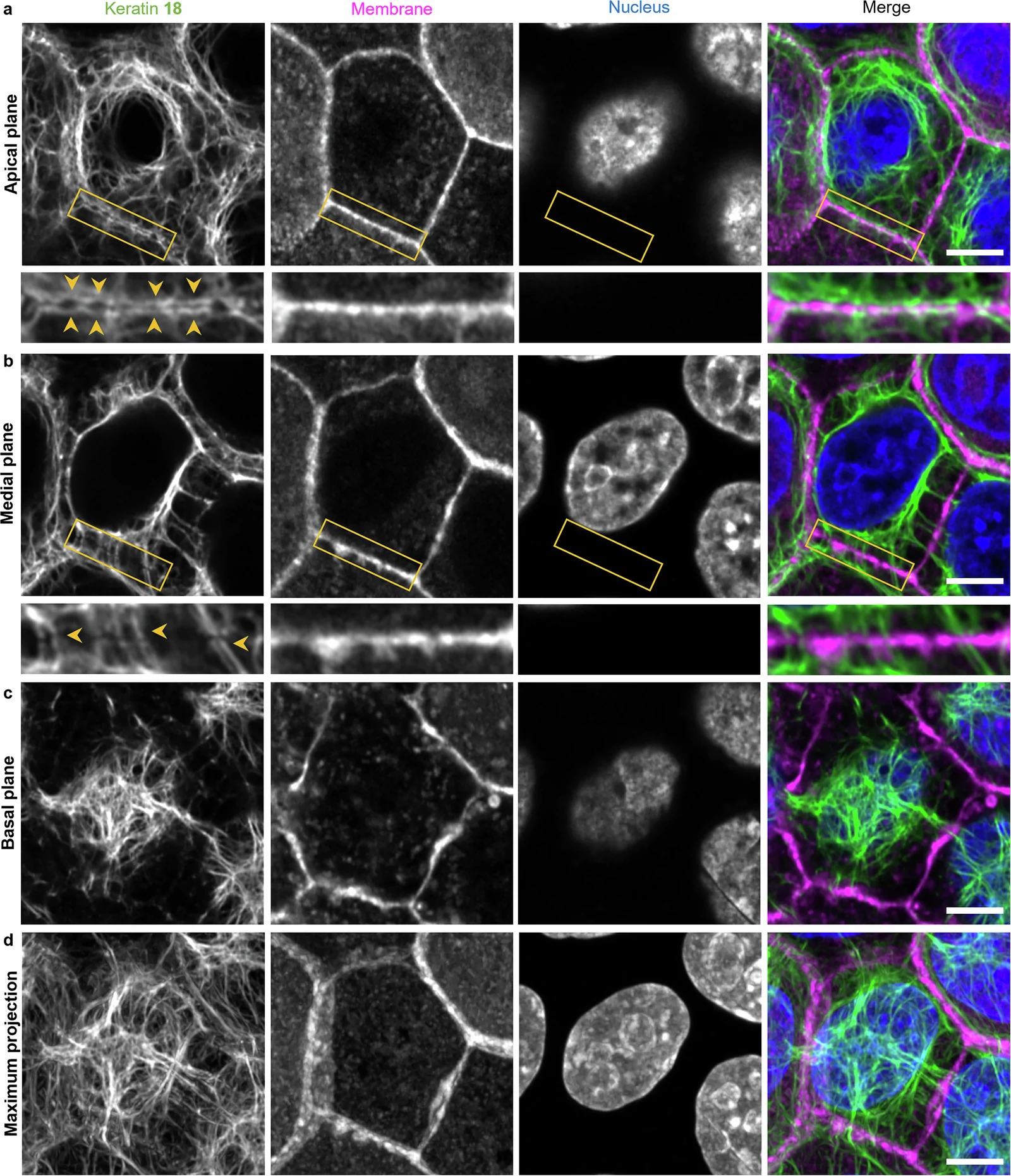
Rim-and-spoke organization in relaxed monolayers. Fixed MDCK monolayer expressing keratin-18-GFP (green) and CAAX-mCherry (magenta) stained to label nuclei with Hoechst (blue). **a**, Apical plane. Inset: Zoom in on the keratin rim lining the plasma membrane on both sides of the cell-cell junction (yellow arrows). **b**, Medial plane. Inset: Zoom in on the keratin spokes aligning perpendicular to the membrane on both sides of the cell-cell junction (yellow arrows). **c**, Basal plane. **d**, Maximum projection of the whole cell showing that the nucleus is fully surrounded by a keratin mesh. Scale bar 5 µm.

We first studied epithelial domes that form spontaneously in MDCK monolayers due to the osmotic pressure generated by directional ion pumping^31,32^. These domes undergo autonomous cycles of inflation and deflation lasting several hours and reaching areal strains of 200–300%. Using micropatterning techniques, we generated arrays of spontaneous domes with a defined footprint radius^19^ and fixed them to visualize the spatial distribution of F-actin and keratin-18 filaments (Fig. 1a and Extended Data Fig. 1a). Although the distribution of F-actin was largely cortical in all cells, keratin displayed two markedly distinct organizations depending on the extent of stretching. Cells within the unstretched monolayer showed a prominent keratin mesh enveloping the nucleus that was connected to the cell surface through thin and short bundles (Fig. 1b and Extended Data Fig. 2). These unstretched cells also displayed a thin keratin network lining the cell cortex, consistent with the rim-and-spoke arrangement commonly reported in epithelial cells^33,34^. By contrast, stretched cells within the dome had a star-like arrangement composed of a dense central node and long, thick bundles radiating towards the cell periphery (Fig. 1b). Each bundle was mirrored by a corresponding bundle in the neighbouring cell, leading to the emergence of a supracellular percolated network of interconnected bundles. This network defines supracellular clusters bounded by transitional cells that exhibited partial bundling restricted to specific subcellular regions.

**Extended Data Fig. 3:**
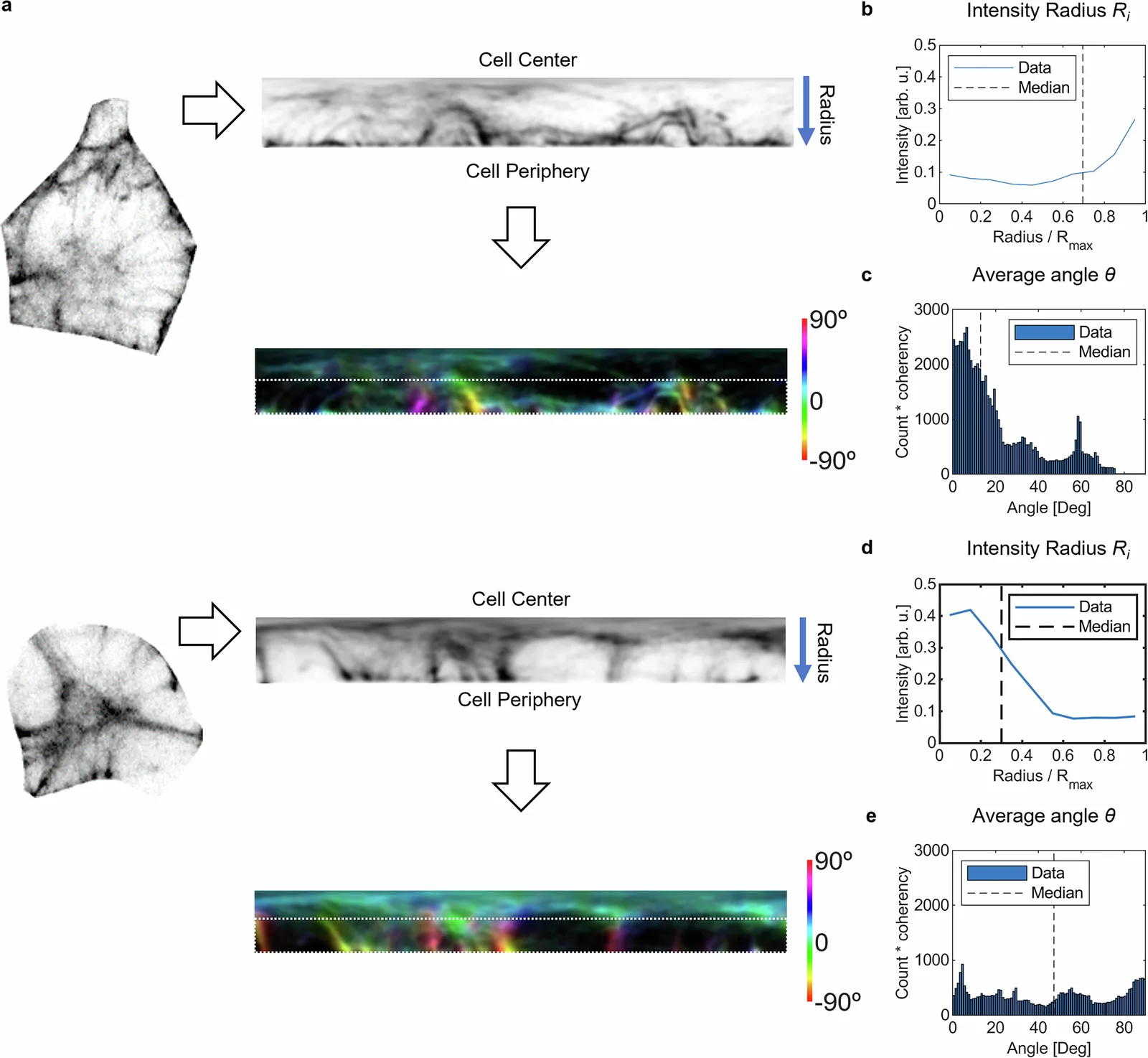
Calculation of Ri and θ. **a**, First, the keratin signal of the cell is transformed into polar coordinates. Second, the mean intensity is measured as a function of the radius, rescaled by the maximum radius R_max_. The median of this curve is R_i_ (**b,d**). Third, the outer half (dotted area) of the cell is used to calculate the direction of keratin features in every pixel. From this, the distributions of angles weighted by their respective coherency is determined. The median of this curve is the average angle θ (**c,e**). For more details see the method section. Source data

**Extended Data Fig. 4:**
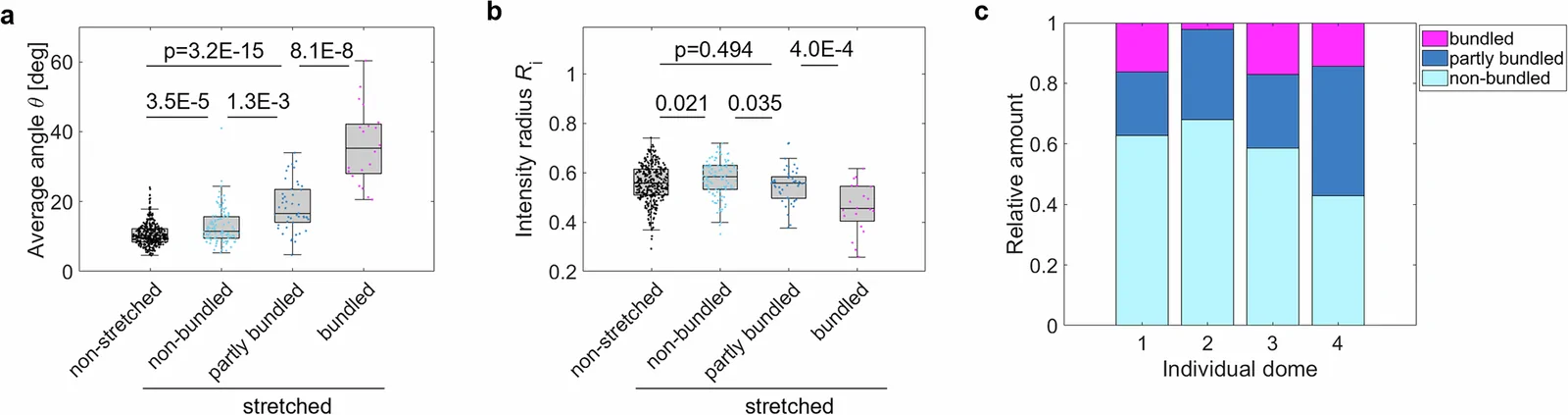
Bundling state of stretched and non-stretched cells in spontaneous domes. **a**, Average angle θ of non-stretched cells compared to stretched cells (non-bundled, partly bundled, and bundled cells). **b**, Intensity radius R_i_ of non-stretched cells compared to stretched cells (non-bundled, partly bundled, and bundled cells). **c**, Relative amounts of non-bundled, partly bundled and bundled cells in 4 fixed domes. Boxes indicate the median and interquartile range, and whiskers extend to minimum and maximum values in (**a,b**). n = 509 cells from 4 domes in (**a,b**). n_1_ = 43 cells, n_2_ = 47 cells, n_3_ = 41 cells, n_4_ = 28 cells in (**c**). Source data

To assess the bundling state of each cell from keratin images, we defined two metrics (Fig. 1c). One is the intensity radius *R*_i_, which reflects the radial distribution of keratin within the cell. Values close to 1 indicate a predominantly peripheral localization, whereas values near 0 indicate a central localization. The second metric is the average angle *θ*, which quantifies the orientation relative to the cell tangent for keratin filaments in the outer half of the cell. This metric approaches 90° for filaments organized radially and 0° for those arranged circumferentially (Fig. 1c and Extended Data Fig. 3). Non-bundled cells displayed high values of *R*_i_ and low values of *θ*, which highlights that filaments accumulated close to the cell junctions and arranged parallel to it. Conversely, cells forming star-like bundles showed low values of *R*_i_ and high values of *θ*, reflecting the central accumulation of keratin and the radial orientation of the bundles. Partly bundled cells displayed intermediate values of *θ* but values of *R*_i_ like those for non-bundled cells, which reflects the absence of a central knot (Fig. 1c–f and Extended Data Fig. 4a,b). All unstretched cells outside the dome were in the non-bundled configuration, whereas in the dome, 29 ± 10% (mean ± s.d.) were partly bundled and 12 ± 7% were fully bundled (Extended Data Fig. 4c). The analysis of these metrics across all cells in the domes established a clear dependence between cell area and its bundling state. Indeed, by plotting *θ* versus cell area, we found that small cells were generally non-bundled, large cells were bundled and intermediate-sized cells were partly bundled (Fig. 1g). This result prompted us to study the mechanisms underlying bundle formation and their dynamics.

### Dynamics of the star-bundling transition

**Fig. 2:**
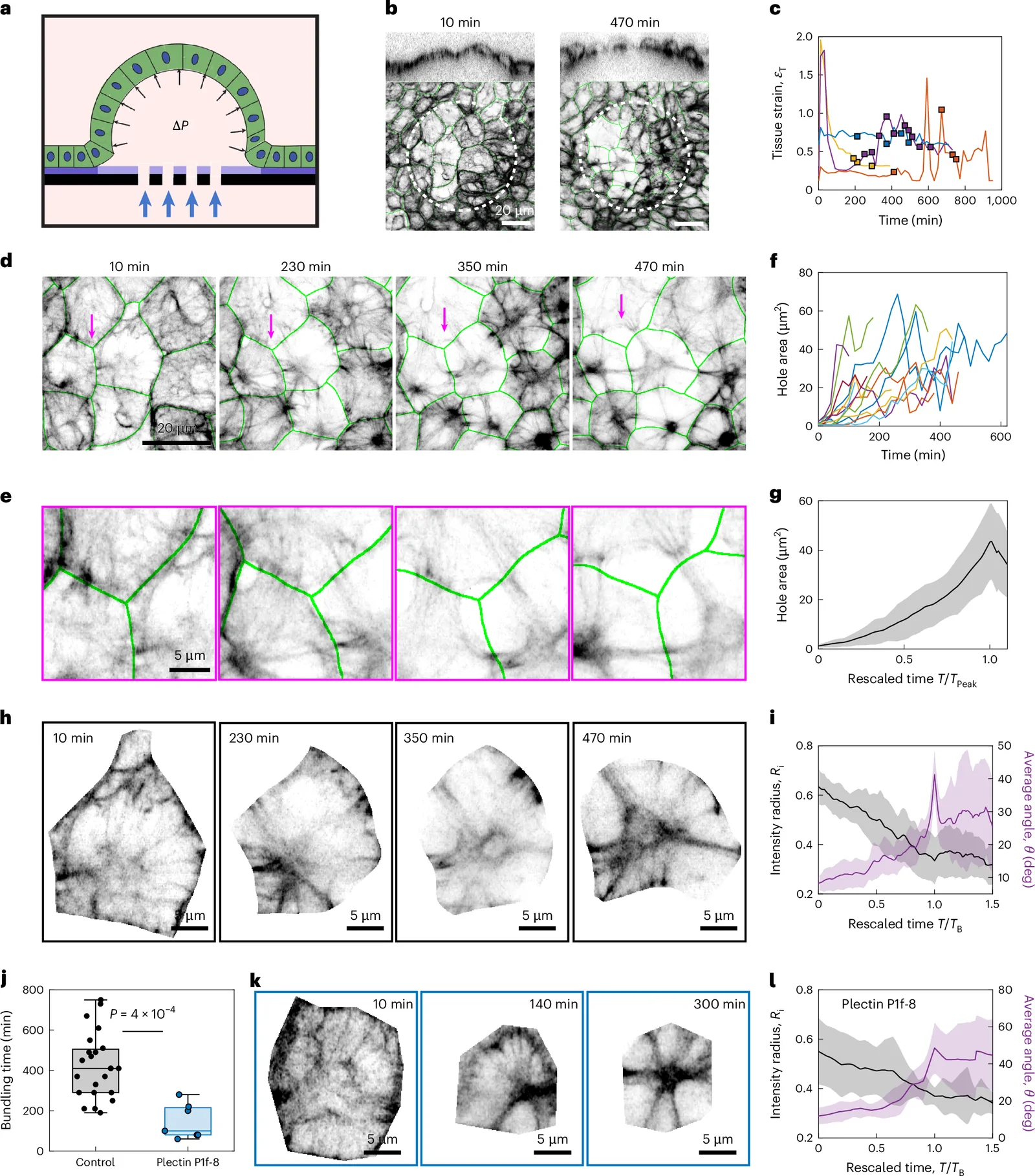
Bundling dynamics in directed domes. **a**, Schematic of the microfluidic device showing the porous membrane (black) coated with high (dark violet) and low (light violet, footprint) fibronectin levels. Hydrostatic pressure applied from the basal side (blue and black arrows) causes the cell monolayer to delaminate at the footprint, resulting in the formation of a dome. **b**, Maximum intensity projections and side views of a directed dome of keratin-18–GFP cells. The dome footprint is marked with a dotted white circle, and segmented cell borders are marked in green. **c**, Time evolution of a tissue strain for four independent domes. Squares mark each bundling event, defined as an individual cell crossing the bundling threshold. **d**, Time sequence of cropped images of a directed dome. Arrows indicate the formation of a keratin-depletion zone at a tricellular junction. **e**, Zoomed-in views of the tricellular junction marked in **d**. **f**, Time evolution of the area of individual depletion zones (*n* = 13 cells). **g**, Mean area of the depletion zone as a function of time rescaled by the peak time *T*_Peak_ (*n* = 13 cells). **h**, A representative individual cell undergoing the bundling transition. **i**, Mean intensity radius *R*_i_ (grey) and mean average angle *θ* (violet) plotted against time rescaled by the bundling time *T*_B_ (*n* = 23 cells). **j**, Bundling time for control cells (*n* = 23) and cells overexpressing plectin P1f-8–BFP (*n* = 7). Boxes indicate the median and interquartile range, and whiskers extend to the minimum and maximum values. **k**, A representative individual cell overexpressing plectin P1f-8–BFP and undergoing the bundling transition. **l**, Same as **i** but for P1f-8–BFP cells (*n* = 7 cells). Shaded areas in **g**, **i** and **l** represent the standard deviation. Source data

In previous theoretical work, we hypothesized that bundling could be explained as a topological phenomenon in an entangled network of filaments connected to laterally mobile desmosomes, such that the straightening of keratin filaments upon stretching is ultimately hindered by a self-organized knot^35^. To test this hypothesis, we developed a microfluidic device that formed domes with a defined geometry and with dynamic control of inflation (Fig. 2a and Extended Data Fig. 1b). This device consists of two perpendicular channels separated by a thin polydimethylsiloxane (PDMS) layer containing evenly spaced holes with a diameter of 80 µm that define the dome footprint. This PDMS layer is covered with a permeable polycarbonate membrane featuring 400-nm-sized pores that allow the passage of liquid but not of cells. Using a photopatterning technique (PRIMO^36^), the polycarbonate membrane was coated with a high concentration of fibronectin outside the footprint and a low concentration of fibronectin inside it. This enabled the seeding of cells and the subsequent formation of a confluent monolayer on the fibronectin-coated side. Hydrostatic pressure was then applied by injecting fluid beneath the membrane, resulting in delamination of the monolayer only at the low adhesion footprint. With this approach, we formed directed epithelial domes of defined geometry and controlled their luminal pressure over time (Fig. 2a and Extended Data Fig. 1b).

**Extended Data Fig. 5:**
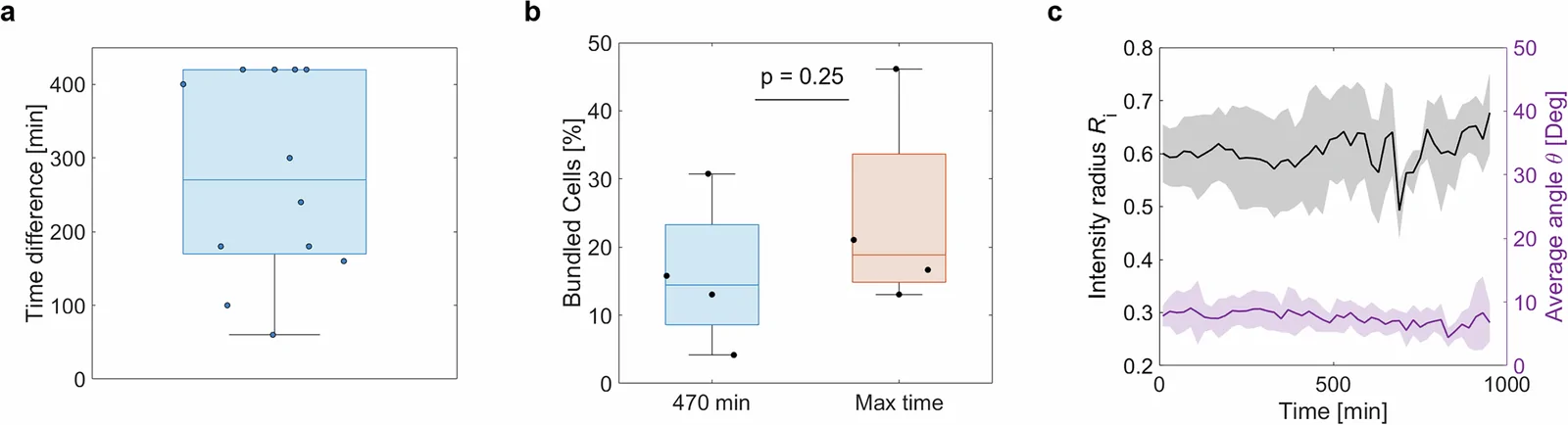
Bundling dynamics inside and outside directed domes. **a**, Time difference between the appearance of keratin depleted zones and the first adjacent cell crossing the bundling threshold (n = 13 cells). **b**, Percentage of bundled cells within the dome after 470 min and after the maximum inflation time of each dome. Significance was assessed with paired-sample t-test. **c**, Mean intensity radius R_i_ (grey) and mean average angle θ (violet) of non-stretched cells outside of the dome footprints (n = 16 cells). Shaded areas represent standard deviation. Boxes indicate the median and interquartile range, and whiskers extend to minimum and maximum values in (**a,b**). Source data

Using this device, we applied a constant pressure of 200 Pa and performed confocal time-lapse imaging of keratin 18 tagged with green fluorescent protein (keratin-18–GFP) and a cell membrane marker for several hours (Fig. 2b). Despite the application of constant pressure, the dome strain fluctuated substantially over time, probably due to discrete cell division and extrusion events as well as transient leaks at cell–cell junctions. (Fig. 2c). Contrary to our expectations, keratin bundling did not occur immediately on stretching (Fig. 2b,d and Supplementary Video 1). Instead, only after tens of minutes did we observe the slow formation of keratin-depleted zones at tricellular junctions (Fig. 2d,e and Supplementary Video 2). These zones gradually grew over time, reaching an average area of 43 ± 15 µm^2^ within 4.5 ± 2.3 h. Keratin filaments depleted from these zones progressively moved towards the perpendicular bisector of each cell–cell junction, ultimately leading to the star-shaped geometry characteristic of stretched cells (Fig. 2d–g). The star-shaped geometry formed at least 60 min and up to 420 min after keratin-depleted zones had started to increase in size (Extended Data Fig. 5a). Although keratin-depleted zones appeared as a gap in the monolayer, the actin cortex and cell–cell junctions remained continuous, indicating that epithelial integrity was preserved (Fig. 1b). We, thus, conclude that tricellular junctions are the nucleation sites of a structural reorganization of the keratin cytoskeleton, which directly initiates the bundling transition.

**Extended Data Fig. 6:**
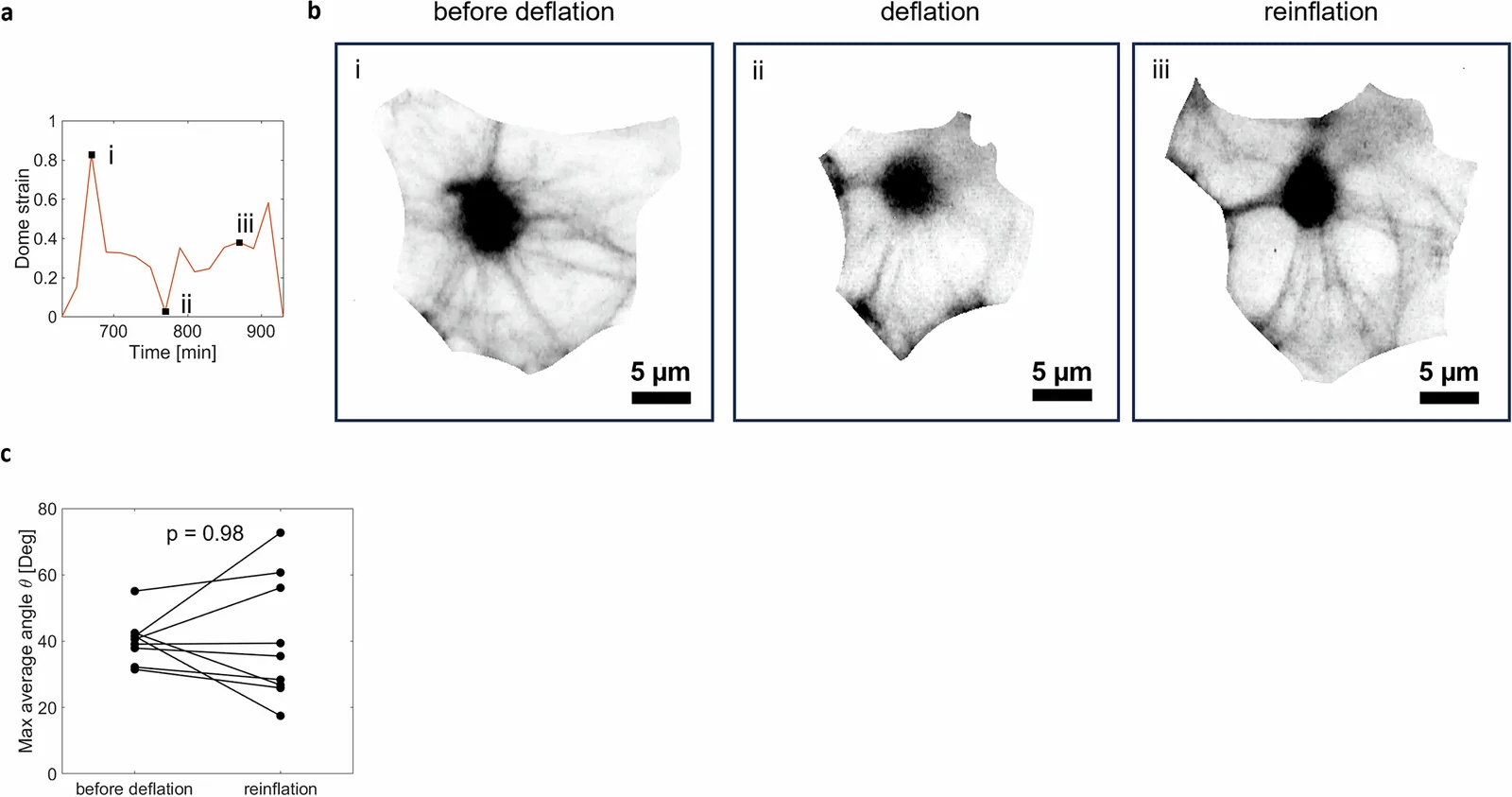
Bundles persist during deflation and reinflation. **a**, Dome strain of a directed dome during a spontaneous deflation and reinflation cycle. Black squares mark the time points displayed in (**b**). **b**, Maximum projection of an individual cell expressing keratin-18-GFP showing that the star-like bundle structure persists during dome deflation and reduction in cell area. **c**, Maximum average angle θ of cells that were bundled before a dome deflated and the maximum θ after the dome reinflated again. Significance tested with paired-sample t-test (n = 9 cells from 3 domes). Source data

To quantify the dynamics of keratin bundling, we computed the time evolution of *θ* and *R*_i_ and defined the bundling transition using a threshold based on these two metrics (Methods). The average bundling time was 424 ± 162 min, with substantial variability between cells (Fig. 2j). After 470 min, 16 ± 11% of cells in the dome were bundled, increasing to 24 ± 15% at the latest time point of inflation (Extended Data Fig. 5b). To systematically analyse the dynamics of bundle formation across the cell population, we plotted *θ* and *R*_i_ as functions of time rescaled between 0 and 1, where 0 corresponds to the onset of pressure application and 1 to the completion of the bundling transition. For cells undergoing the bundle transition, there was a gradual decrease of *R*_i_ from predominantly peripheral (>0.5) to central (<0.5), whereas *θ* increased smoothly (Fig. 2h,i). Notably, *θ* showed a sharp rise shortly before the bundling threshold was crossed, thus revealing accelerated cytoskeletal dynamics just before the transition. After crossing the threshold, *θ* remained high and *R*_i_ remained low, showing that cells remained in the bundling state. Even after spontaneous dome deflation and reinflation episodes, bundling was not lost (Extended Data Fig. 6), indicating that the bundling transition was irreversible within the timescales of our study. Cells outside the dome footprint, and thus not stretched, displayed constant values of both *θ* and *R*_i_, demonstrating that stretching is necessary for triggering the bundle transition (Extended Data Fig. 5c).

In summary, our findings indicate that the star-bundling transition is not an immediate reaction to the increase in cell area but rather a slow adaptation to stretching over hundreds of minutes. Therefore, the previously reported strain-stiffening behaviour produced by keratin in epithelial cells at short timescales (<1 min)^17^ is independent of the bundling transition.

### Bundling is hindered by the actin cortex

**Extended Data Fig. 7:**
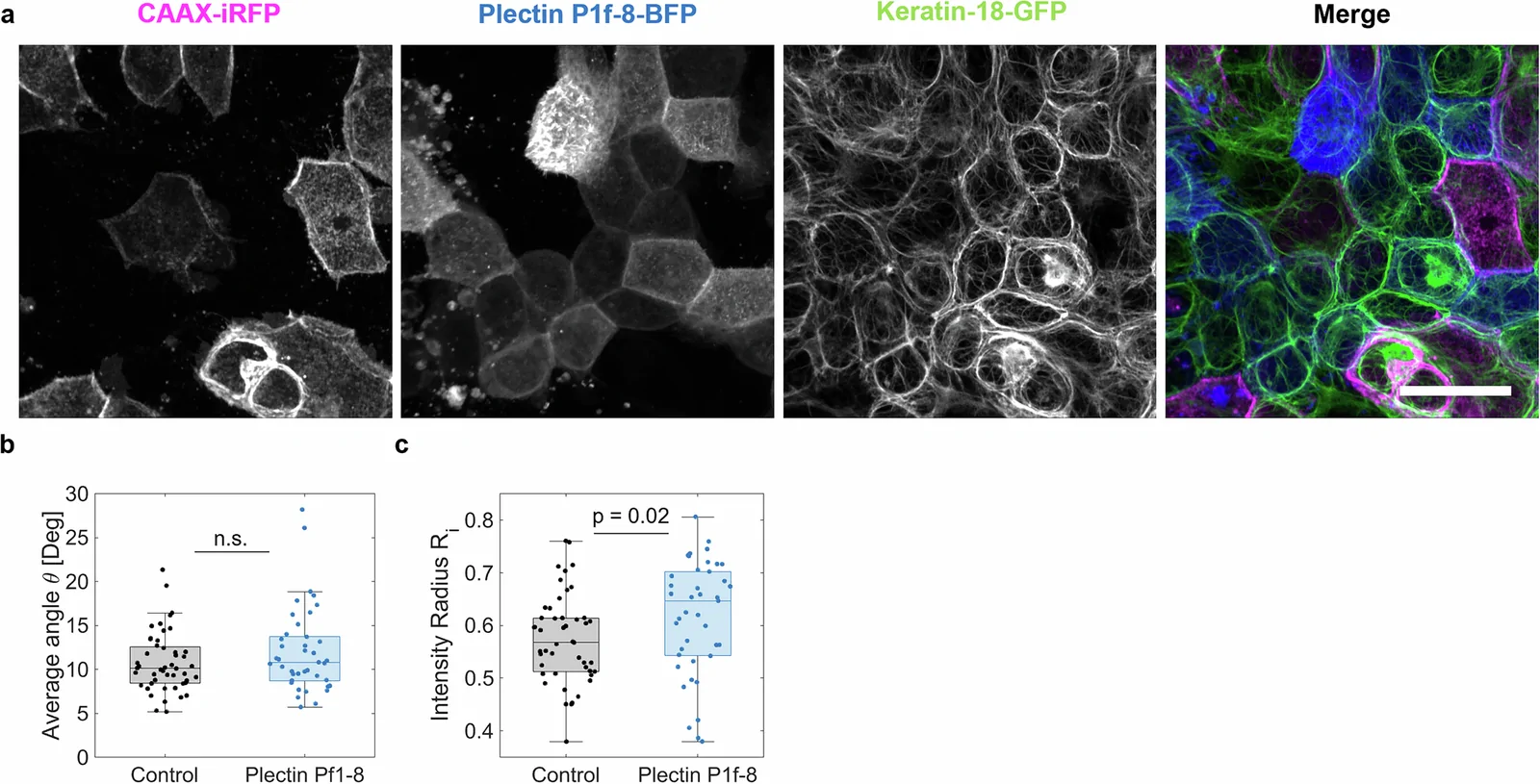
Plectin P1f-8-BPF cells display a similar keratin structure as control cells. **a**, Fixed image of a mixed population of keratin-18-GFP (green) cells expressing either CAAX-iRFP (magenta, control) or P1f-8-BFP (blue). **b**, Average angle θ of control cells and cells expressing P1f-8-BFP. **c**, Intensity radius R_i_ of control cells and cells expressing P1f-8-BFP. Measurements in (**b,c**) are based on clusters of the same cell types in mixed populations in fixed simples. Boxes indicate the median with lower and upper quartile and whiskers extend to minimal and maximal values. (n = 48 control cells, n = 40 P1f-8-BFP cells). Differences in θ are non-significant whereas differences in R_i_ are statistically significant but small. Scale bar 20 µm. Source data

We next investigated the mechanisms underlying the slow dynamics of the bundling transition. An appealing candidate is the interaction between the intermediate filaments and the actin cytoskeleton, which is well known to influence the cytoskeleton architecture^37–39^. To study the role of this interaction, we targeted plectin, the main crosslinking protein between keratin and actin^22,40,41^. Specifically, we induced a loss of plectin function by overexpressing a truncated version of the plectin isoform P1f tagged with blue fluorescent protein (P1f-8–BFP). This mutant retains the actin-binding domain but lacks the keratin-binding domain, thereby it acts as a dominant negative mutant that weakens the specific interaction between the actin cortex and the keratin cytoskeleton^38^. In MDCK cells, P1f-8–BFP localizes mainly at the cell cortex (Extended Data Fig. 7).

**Extended Data Fig. 8:**
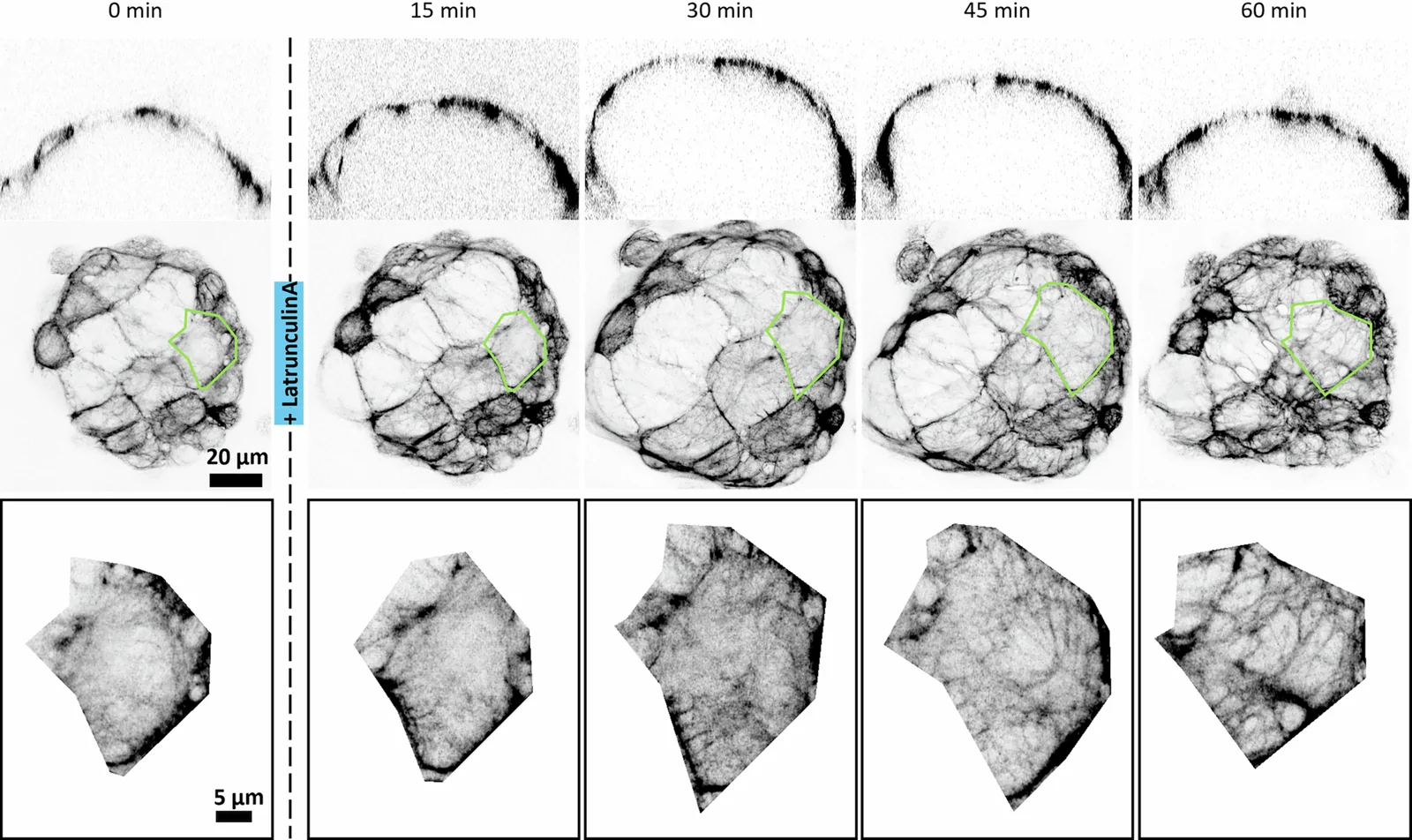
Latrunculin A treatment causes fast keratin bundling. Top**:** Side view of an inflated dome treated with Latrunculin A. Middle: Maximum projection of the upper part of the dome with one cell segmented in green. Bottom: Individual segmented cell from the same dome. Representative images for 6 domes.

In the absence of stretching, cells expressing P1f-8–BFP and control cells exhibited comparable keratin organizations (Extended Data Fig. 7). Upon stretching, the P1f-8–BFP cells underwent a bundling transition like that observed in control cells (Fig. 2k,l). However, the average bundling time was significantly reduced to 146 ± 68 min (Fig. 2j). This near threefold decrease establishes that the keratin–actin interaction is the main determinant of the bundling rate. To investigate the role of the actin cortex during the bundling transition, we treated control MDCK domes with 1 µM latrunculin A 15 min after dome formation, which caused a rapid disassembly of actin filaments. This perturbation resulted in a quick reorganization of the keratin cytoskeleton into a more bundled network in less than 60 min, although the characteristic star-like bundle organization observed in untreated cells was not fully recapitulated (Extended Data Fig. 8). These experiments establish that actin–keratin interactions are the main determinant of the bundling transition during stretching.

### A computational model captures the bundling transition and predicts nuclear uncaging

Aiming to recapitulate the dynamics of the bundling transition, we expanded our previous computational framework, which was constrained to a two-dimensional geometry and did not account for keratin interactions with the actin cytoskeleton or the nucleus^35^. We developed a customized version of the open-source simulation suite Cytosim^42^, which enables stochastic simulations of cytoskeletal fibres governed by Brownian dynamics (Supplementary Note). Our new model simulates a representative cell comprising flexible and entangled keratin fibres whose ends are attached to the cell periphery via laterally mobile bonds representing desmosomes. Fibres interact with each other through steric repulsion forces, which prevent filament crossing, and short-range adhesive forces, which promote bundling.

**Fig. 3:**
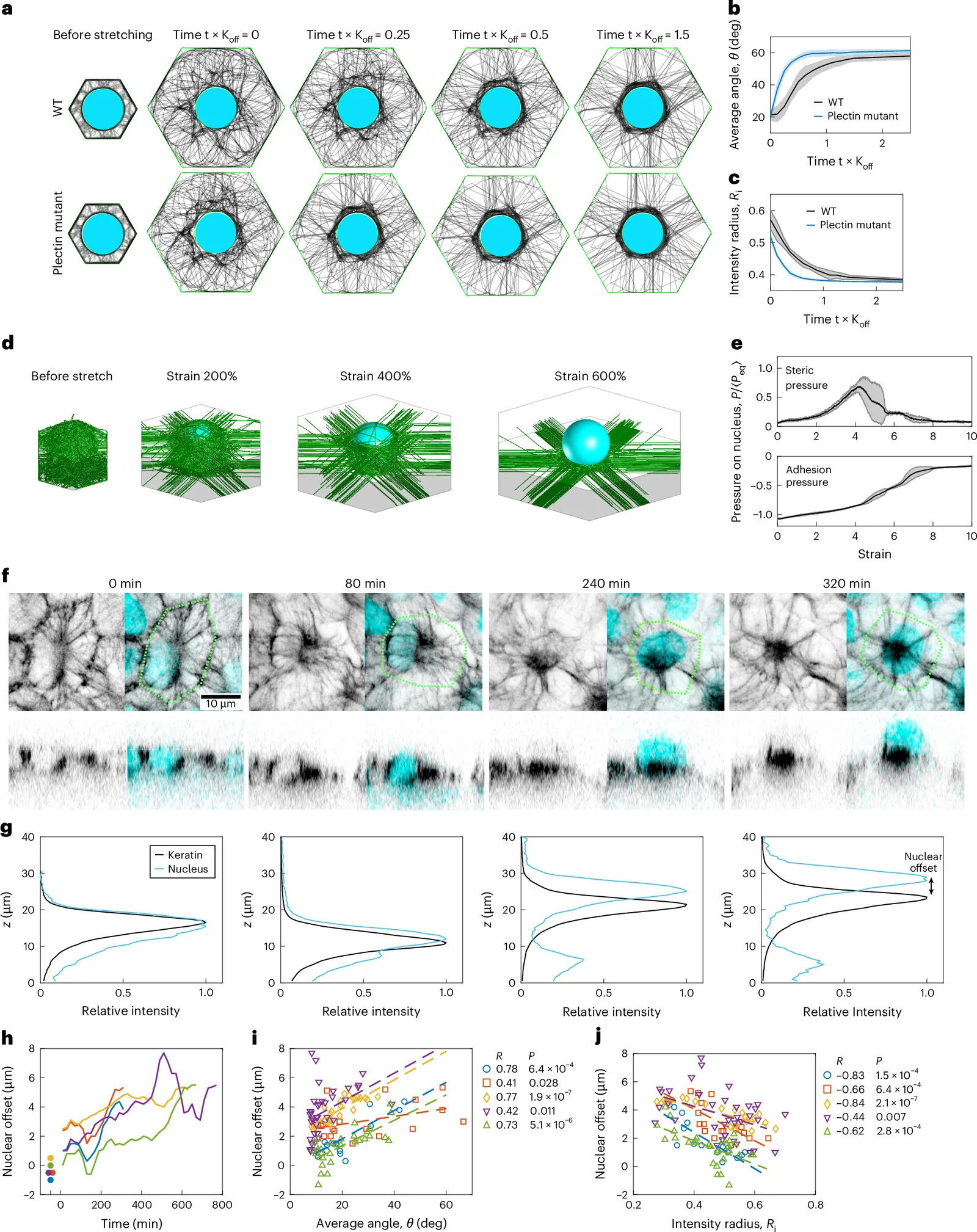
The nucleus escapes from the keratin cage during bundling. **a**, Top view of a 2.5D simulation with keratin filaments (black), cell boundaries (green) and the nucleus region (cyan)**. b**, Mean simulated average angle *θ* plotted against time, reported as mean ± s.d. of *n* = 8 model realizations. **c**, Mean simulated intensity radius *R*_i_ plotted against time, reported as mean ± s.d. of *n* = 8 model realizations. **d**, Isometric views of a 3D simulation with keratin (green) and the nucleus (cyan). **e**, Mean simulated pressure on the nucleus plotted against strain in the 3D simulation, reported as mean ± s.d. of *n* = 7 model realizations and with pressure values normalized with respect to the time average of their sum during the equilibration phase preceding cell stretching. **f**, Maximum intensity projections (top row) and side views (bottom row) of a MDCK keratin-18–GFP cell (grey) showing the nucleus (SPY-DNA, cyan) and cell border (green dotted line). **g**, Normalized intensity of the keratin and nuclear signals along the *z* coordinate. **h**, Nuclear offset plotted against time. Dots indicate the values in the relaxed monolayer before dome inflation. Different colours represent different cells. **i**, Nuclear offset plotted against the average angle *θ*. **j**, Nuclear offset plotted against the intensity radius *R*_i_. Dashed lines in **i** and **j** indicate linear fits. *n* = 5 cells in **h**–**j**. *R* values are Pearson’s correlation coefficient. WT, wild type. Source data

**Extended Data Fig. 9:**
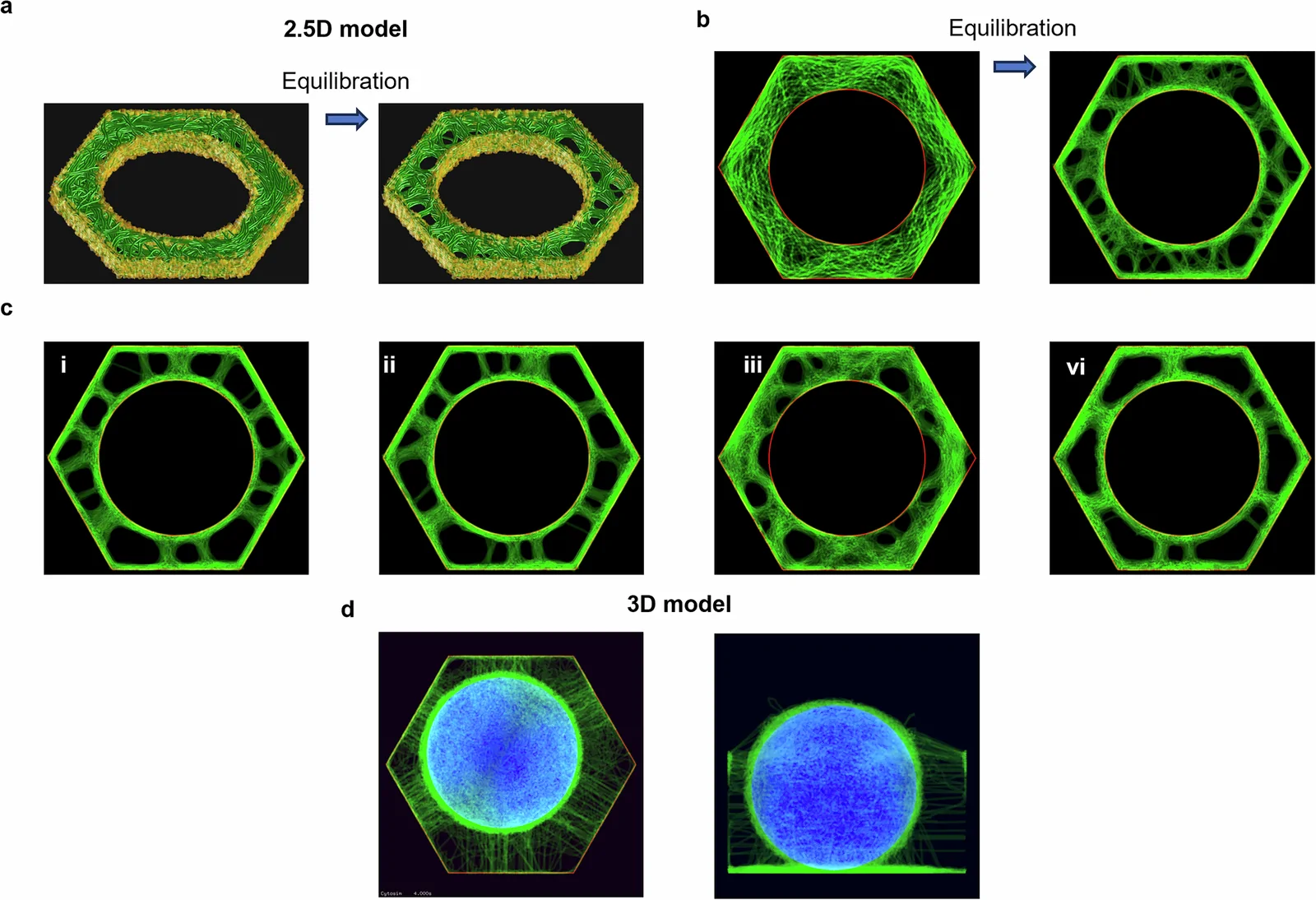
Rim-and-spoke arrangement in simulations. **a**, Model view before and after equilibration showing the emergence of rim-and-spoke structure as the result of keratin (green) interaction with cortical actin and nucleus via discrete linkers (yellow) (parameters:${K}^{-}/{K}^{+}=0.1$, ${d}_{b}/{a}_{0}=0.1$). **b**, Top view of the model before and after equilibration. **c**, Alternative parameters for rim-and-spoke formation: **i)** ${K}^{-}/{K}^{+}=1$, ${d}_{b}/{a}_{0}=0.1$ **ii)** ${K}^{-}/{K}^{+}=10$, ${d}_{b}/{a}_{0}=0.1$ **iii)** ${K}^{-}/{K}^{+}=0.1$, ${d}_{b}/{a}_{0}=0.05$ **iv)** ${K}^{-}/{K}^{+}=10$, ${d}_{b}/{a}_{0}=0.2$. **d**, Top and side view of a 3D model after equilibration showing keratin (green), cell boundaries (red) and nucleus (blue).

Within this computational framework, we first implemented a 2.5-dimensional (2.5D) simulation of the region around the medial plane of the cell (Fig. 3a and Extended Data Fig. 9a,b), where the rim-and-spoke organization is better observed experimentally. In this 2.5D model, the nucleus is represented by a cylindrical region that cannot be penetrated by keratin fibres. Keratin interactions with the nuclear surface and the cell cortex were modelled by uniformly distributing many discrete stochastic linkers on both surfaces. These linkers represent plectin proteins that connect keratin with the actin cortex by binding to actin filaments and with the nuclear envelope by binding to nesprin-3^43^. In the model, the linkers bind fibres stochastically with binding rate ${K}^{+}$ and unbind as slip bonds with a force-dependent rate. As the nucleus moves or the cellular surfaces deform, the position of these linkers evolves accordingly. Notably, the introduction of such linkers into an initially disordered keratin network was sufficient to drive its spontaneous reorganization into a rim-and-spoke architecture after an equilibration period ${t}_{\mathrm{eq}}\gg 1/{K}^{+}$ (Extended Data Fig. 9c). The emergence of such a characteristic arrangement was robust across a range of parameter choices, indicating that the rim-and-spoke topology is self-organized and arises generically from interactions between keratin fibres and the intercellular and nuclear ‘sticky’ surfaces bounding the network.

Next, we subjected the model to in-plane loading by applying a stretch-and-hold protocol. In the control case, stretching led to a gradual loss of the rim, the formation of load-bearing radial bundles and an accumulation of fibres around the nucleus, which we quantified by plotting *θ* and *R*_i_ as in the experiments (Fig. 3a–c). To test the effect of the plectin loss of function, we reduced the number of discrete linkers by 50%. In this case, stretching led to the same characteristic reorganization of the keratin structure but on an accelerated timescale (Fig. 3a–c). This confirmed that keratin–actin interactions play a key role in modulating the timescale of the bundling transition. However, the simulation never reached the full star-like organization of the keratin network due to the geometric obstacle posed by the nucleus in the 2.5D model.

To address this limitation and better investigate the interaction between keratin and the nucleus, we developed a second set of models that were three-dimensional (3D). In these models, the cell is a hexagonal prism, and the nucleus is a rigid sphere with translational degrees of freedom. The nucleus interacts sterically with entangled keratin fibres attached to the side surfaces of the cell via laterally mobile links (Extended Data Fig. 9d). To account for the apical–basal asymmetry of epithelial cells, we introduced a confining potential for the nucleus on the bottom and side walls of the cell membrane but not on its apical surface. Given the computational complexity of the 3D models, we simplified interactions between keratins and other cytoskeletal components by introducing short-range adhesive potentials on the nuclear shell and on the bottom and side cell surfaces. We first confirmed that this modelling approach also captures the rim-and-spoke keratin arrangement (Extended Data Fig. 9d). We then gradually stretched the model at a constant strain rate. As the strain was increased, keratin reorganized into a star-like bundle structure and keratin fibres apically covering the nucleus were progressively lost. This was followed by a rapid escape of the nucleus from the keratin cage as the perinuclear network slid basally to form the central knot (Fig. 3d and Supplementary Video 3).

We then wondered about the physical forces exerted by the keratin fibres on the nucleus during this uncaging process. Thus, we computed two contributions to nuclear pressure: the adhesion pressure, which results from the adhesive interaction between the nucleus and the keratin network, and the steric pressure, which arises because the keratin fibres are physically excluded from the nucleus. Before stretching, the steric pressure was nearly zero, whereas the adhesion pressure was negative, reflecting the tension of the ‘spoke’ fibres pulling on the nucleus (Fig. 3e). Upon stretching, the pulling forces generated on the nucleus by adhesion progressively diminished due to the nucleus disengaging from the keratin network. By contrast, the steric pressure increased sharply as the fibres tightened against the nucleus and then dropped abruptly after nuclear escape (Fig. 3e). Counter-intuitively, this indicates that pulling on the tissue results in nuclear compression, which eventually pushes the nucleus out of its surrounding keratin cage.

### The nucleus is pushed out of its keratin cage

To test this prediction, we first inspected the fixed spontaneous domes (Fig. 1b). As predicted, we found that the star-like bundle structures are localized on the basal side of the cell whereas the nucleus is positioned on top of this basal structure and is surrounded by only a residual amount of keratin. This stands in stark contrast to non-bundled cells, where the nucleus resides in the medial plane and is surrounded by a prominent keratin cage in all three dimensions (Fig. 1b and Extended Data Fig. 2).

To understand the dynamics of this drastic structural transition, we performed simultaneous live confocal imaging of keratin-18–GFP and the nucleus (labelled with SPY-DNA) at constant dome pressure (Fig. 3f). At the onset of inflation, before bundling, the peaks of the fluorescence intensity of keratin and DNA were roughly colocalized along the *z* axis, indicating that the nucleus was fully embedded in its keratin cage. With time, the nucleus progressively escaped from the cage, as revealed by an increasing offset between the keratin and DNA peaks (Fig. 3g,h). Eventually, the nucleus fully exited the cage, giving rise to the formation of the central knot characteristic of star-like bundled cells (Fig. 3f–h). To study the coupling between the bundling transition and nuclear escape, we plotted the nuclear offset with respect to *θ* and *R*_i_. We found that the offset is strongly correlated with both metrics across individual cells, indicating a robust link between keratin bundling and nuclear uncaging (Fig. 3i,j). Together, our computational model and experiments paint a physical picture whereby an increase in the tension in keratin fibres generates a compressive stress on the nucleus, which is ultimately squeezed out of its cage.

### Supracellular clusters emerge through nucleation and growth

We finally investigated the supracellular dynamics of the bundling transition. Fixed images of spontaneous domes show that bundled cells arrange in multicellular clusters (Fig. 1a,e). These clusters could emerge through different spatio-temporal scenarios. A first possibility is that bundled cells initially form in separate locations of the dome and subsequently move to coalesce into a single cluster. Alternatively, all cells in a cluster could bundle simultaneously to form a supracellular bundled network in a single step. A third scenario is that bundling nucleates in a single cell and progressively propagates by recruiting neighbouring cells into the cluster.

**Fig. 4:**
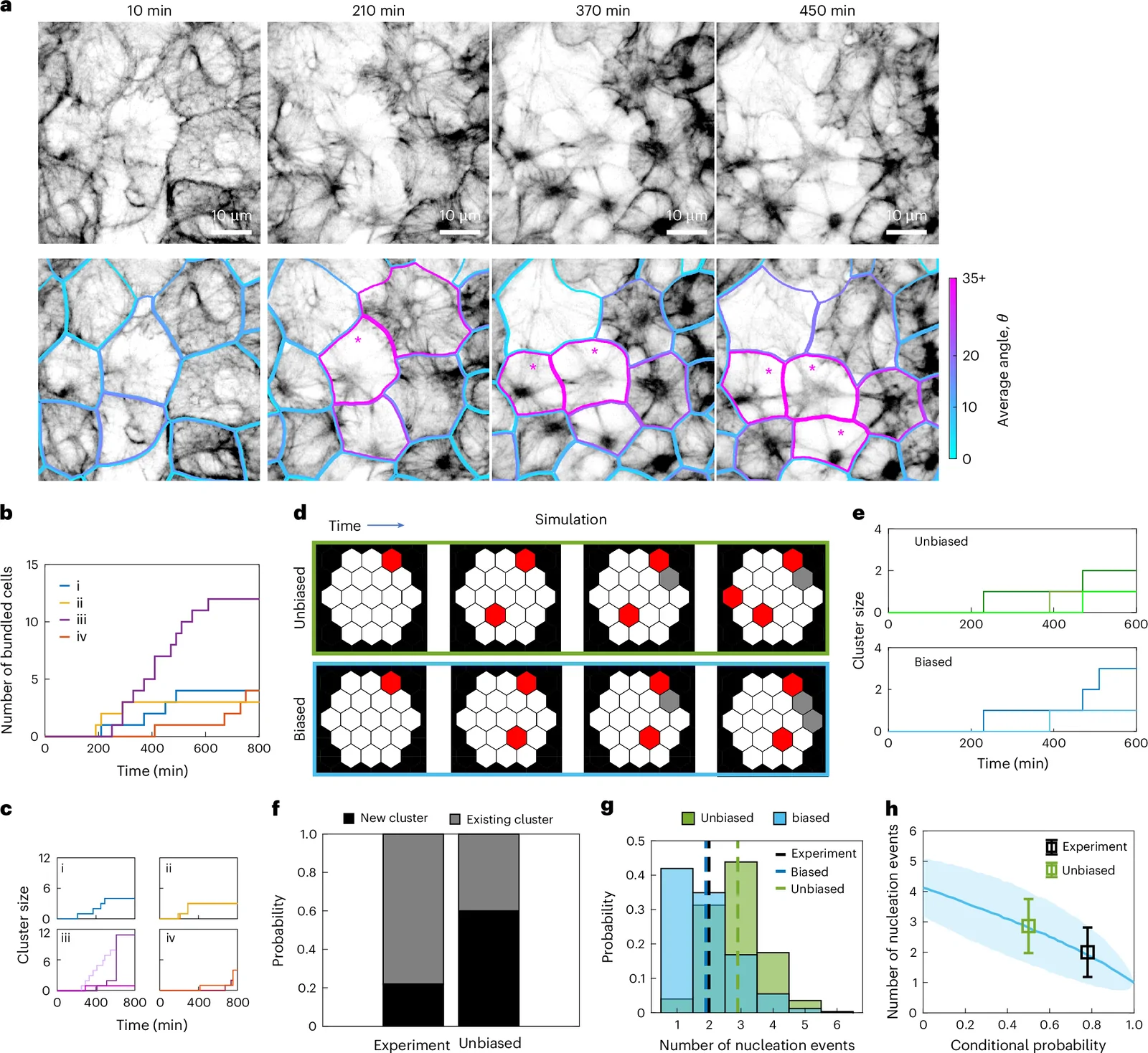
Bundling is a collective process. **a**, Cropped images of MDCK keratin-18–GFP cells in a directed dome. Outlines indicate segmented cell borders colour-coded according to the average angle *θ*. Frames were chosen to display individual cells crossing the bundling threshold sequentially (marked with an asterisk). **b**, Number of bundled cells over time in four domes. **c**, Cluster size over time in four domes. If a dome has several clusters, they are shown with different intensities until they merge. **d**, Examples of sequential bundling events in unbiased and biased simulations. Black lines represent cell borders. Cells starting a new cluster count as a nucleation event and are marked in red. Cells bundling adjacent to an existing cluster are marked in grey. **e**, Cluster size over time in the simulations shown in **d**. **f**, Probability of bundling cells forming a new cluster (black) or joining an existing cluster (grey) 100 min after the first bundled cell in a dome. Data are shown for experiments and for the unbiased simulation. **g**, Probability distribution of the number of nucleation events per dome at the end of the simulation. Dashed lines indicate mean values. *n* = 4,000 simulations each. **h**, Mean number of nucleation events plotted against the conditional probability of joining an existing cluster in biased simulations. The shaded area represents the standard deviation. Data points show the experimental probability (*n* = 4 domes) and the unbiased simulation probability (*n* = 4,000 simulations), represented as mean ± s.d. Source data

To distinguish between these different scenarios, we analysed time-lapse sequences of keratin-18–GFP domes subjected to a step pressure of 200 Pa, which ensured that none of the domes had previously experienced inflation. A representative sequence is shown in Fig. 4a (Supplementary Video 4). In the bottom row of this panel, the cell outline is colour-coded according to the average angle *θ* of each cell, whereas in the top row, the same images are shown without outlines to allow visualization of the cortical keratin rim, which is a principal contributor to *θ*. This sequence shows that, following an initial period without detectable bundling, one cell undergoes a bundling transition whereas some of its neighbours exhibit a progressive increase in *θ*. With time, these neighbouring cells also cross the bundling threshold, leading to the growth of a cluster. This observation indicates that bundling occurs sequentially rather than simultaneously and proceeds through cluster nucleation and growth rather than coalescence.

**Extended Data Fig. 10:**
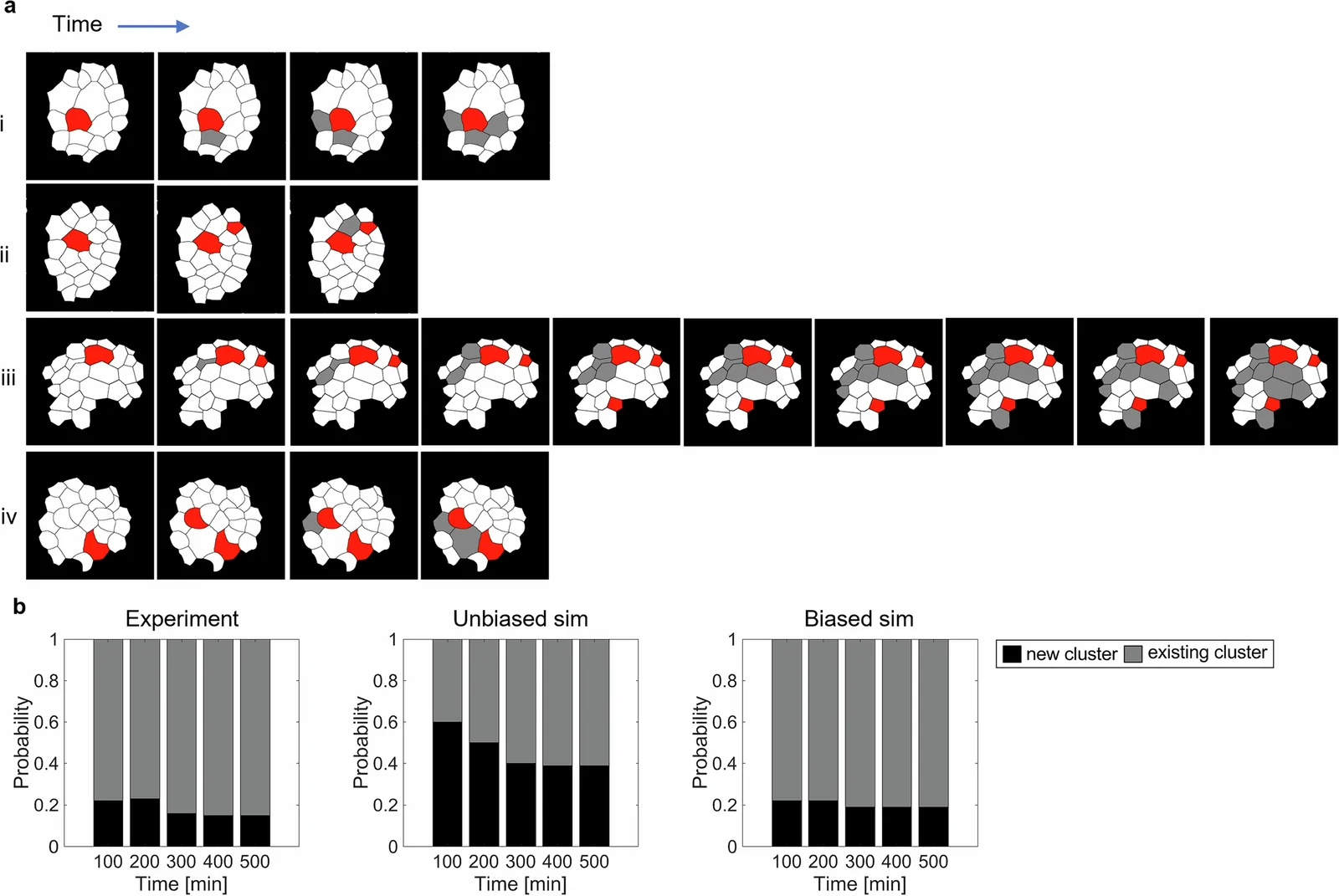
Nucleation event over time. **a**, Sequence of cells crossing the bundle threshold. Cells starting a new cluster count as a nucleation event and are marked in red. Cells being connected to an existing cluster are marked in grey. Roman numbers label different experiments. **b**, Probabilities of bundling cells starting a new cluster or joining an existing cluster over time in experiments, unbiased simulations and biased simulations. The time is in reference to the first cell crossing the bundling threshold. Source data

To substantiate this observation quantitatively, we computed the number of bundled cells per dome over time. This analysis, performed for four different domes, confirmed that bundling is a sequential process that starts approximately after 200 min of inflation and proceeds at an average rate of 1.00 ± 0.65 bundled cells per hour (Fig. 4a,b). In addition, our analysis shows that bundling occurs through the nucleation of clusters (2.0 ± 0.8 nucleation events per dome), which grow over time and eventually merge into larger ones (Fig. 4c and Extended Data Fig. 10a).

These experiments indicate that a cell will more probably bundle if at least one of its neighbours has already bundled. Hence, the growth of a cluster is favoured over the creation of a new one. To ascertain this, we performed a Monte Carlo simulation of dome dynamics under different probabilistic scenarios for a cell to bundle. In our simulations, domes were modelled as two-dimensional hexagonal grids of 19 cells to match the experimental dome size (Fig. 4d). Initially, no cells were bundled. The simulation considers sequential bundling events, in which a cell is randomly selected to switch to the bundled state, and once bundled, it remains in that state. In a first unbiased scenario, all non-bundled cells have the same probability of being selected for switching to a bundled state. The times selected for bundling were taken from the experiments to allow for a direct comparison between simulations and observations (Fig. 4e). We found that within the first 100 min the probability of forming a new cluster was 60%, compared with just 22% in the experiments (Fig. 4f). Both probabilities decreased over time as some clusters had already formed, yet the nearly threefold difference between simulation and experiment persisted (Extended Data Fig. 10b). The average number of nucleation events at the end of the simulation was 2.9 ± 0.9 (dashed green line in Fig. 4g), far above the 2 ± 0.8 observed experimentally (dashed black line in Fig. 4g). Thus, an unbiased scenario in which each cell in the dome has an equal probability of bundling does not account for our experimental results.

This discrepancy led us to consider a second biased scenario in which the probability of bundling is higher if a neighbouring cell has already bundled. As expected, increasing this probability decreased the number of nucleation events in the domes (Fig. 4h). Notably, when each cell was given a 78% chance of being selected if it was adjacent to an existing cluster and a 22% chance if it was not, as measured experimentally (black bars in Fig. 4f), the average number of nucleation events was 1.9 ± 0.9 (dashed blue line in Fig. 4g), which matches the experimental results (dashed black line in Fig. 4g). Together, these simulations support the conclusion that the spatial distribution of bundling events is not completely random but instead arises from a slow nucleation-and-growth mechanism.

## Discussion

In this work we studied the dynamics of the keratin cytoskeleton during the application of a large and sustained stretch. We identified a structural transition that spans several length scales. At the subcellular scale, keratin filaments assemble into thick bundles that radiate from a central knot and bisect cell–cell junctions. At the supracellular scale, cells undergoing this star-bundling transition nucleate clusters that grow in time to form domains. The bundling transition is dynamically regulated by the keratin–actin interaction and is paralleled by the escape of the nucleus from its keratin cage. The transition is irreversible within the timescale of our experiments, even after unstretching. Hence, reversing the star-like bundle structure back to its initial rim-and-spoke configuration would probably require a disassembly of the keratin fibres via phosphorylation, a process that typically takes place during mitosis^44,45^. Interestingly, the bundling transition reflects a geometric transformation between two topological representations of the tissue: the tessellation—the tiling of the plane by polygonal cells in which intermediate filaments initially accumulate at cell–cell junctions—and a dual triangulation formed by supracellular bundles connecting central knots (Supplementary Fig. 1). According to this notion of topological duality, polygons in the tessellation (cells) become vertices in the triangulation (knots), whereas edges in the tessellation (cell–cell junctions) become crossing edges in the triangulation (keratin bundles).

It is widely recognized that the keratin cytoskeleton plays a protective role against large deformations. This role has traditionally been attributed to the strain-stiffening properties of keratin filament networks, which have been linked to structural rearrangements such as keratin bundling^17,19^. However, our findings reveal that the timescales of strain-stiffening and the bundle transition are fundamentally distinct. Although strain-stiffening has been shown to occur over timescales of seconds to minutes^17^, the bundling transition and its supracellular propagation observed in our study span hours to days. The keratin bundling transition should not, therefore, be interpreted as a ‘safety belt’ mechanism that provides strain-stiffening to the tissue under large and fast deformations^46^. Rather, the transition can be viewed as the terminal stage in a progressive time-dependent reorganization of the keratin network, which tends to relax stresses until topologically possible by the formation of the central knot. Our experiments and simulations, in both 2.5D and 3D, indicate that cell geometry and keratin fibre entanglement are sufficient to spatially organize the star-like bundle structure. Although specific interactions, such as those mediated by plectin, modulate the dynamics of the process, they are not required to account for the final geometry of the star-bundled cells.

A notable feature of the structural transition uncovered in this study is the complete escape of the nucleus from its keratin cage. This perinuclear cage is generally thought to play a protective role by shielding the nucleus from large mechanical deformations, in line with observations of single breast epithelial cells, in which a keratin perinuclear network renders the nucleus insensitive to the mechanics of its environment^10^. Our computational results indicate that, for a moderate stretch, the intermediate filament network indeed wraps the nucleus more tightly, thus shielding it from the applied tension. However, upon large and sustained stretching, both our experiments and simulations show that the nucleus exits the keratin cage. Whether this uncaging process compromises or protects the nucleus is a principal question raised by this study. On the one hand, an unshielded nucleus is more vulnerable to mechanical forces. On the other hand, and perhaps counter-intuitively, decoupling the nucleus from the highly stressed cytoskeleton may provide an alternative form of protection by isolating it from force transmission pathways. This hypothesis could be addressed experimentally in the future, for example, by analysing nuclear damage under a prolonged mechanical strain.

Our joint experimental and computational analysis identifies the main determinants that govern the onset and dynamics of the star-bundling transition. The first principal factor is the interaction between keratin and actin, which contributes to slowing down the transition. Consistent with this result, previous work has shown that a loss of function of the actin–keratin crosslinker plectin is sufficient to bundle keratin filaments, even in the absence of mechanical stretching^38^. In addition, large deformations are known to induce thinning of the actin cortex, which potentially reduces the number of actin–keratin interactions, thereby favouring the bundling transition^19^. A second key determinant is the escape of the nucleus from its perinuclear keratin cage. For the star-bundling transition to complete, the keratin network must overcome a mechanical obstacle that arises from the specific and steric interactions with the nucleus. A third potential determinant of the star-bundling transition is the interaction with the extracellular matrix, either directly through hemidesmosomal junctions or indirectly via focal adhesions. Although this interaction cannot be tested in our experimental system as the stretched cells are largely devoid of an extracellular matrix, previous work by our groups has shown that the adhesion of keratin to the laminin through hemidesmosomes affects cytoskeleton mechanics and shields the nucleus from actomyosin-mediated mechanical deformation, which potentially impairs the star-bundling transition^10^. Interestingly, a recent study of the intestinal epithelium showed that, in conditions of low adhesion, actin can adopt a star-shaped morphology like the one observed here for keratin^47^. These results indicate that the star-shaped organization of cytoskeleton filaments might be a general mechanical adaptation, although it is unclear whether the underlying mechanisms and dynamics of actin star formation are analogous to those observed here for keratin.

By uncovering the mechanisms underlying the star-like bundling transition, our study has identified the conditions under which this transition may be relevant in physiology and disease. In early development, tissues are subjected to slow and sustained deformations while often expressing relatively low levels of the extracellular matrix, two factors that favour the bundling transition. It will, therefore, be interesting to investigate whether keratin undergoes partial or complete bundling transitions in developmental systems, such as inflating blastocysts or gastrulating vertebrate embryos^48,49^. In adulthood, the bundling transition might be relevant in glandular organs experiencing large and sustained stretching, such as the bladder or the lactating mammary gland. In the context of disease, keratin bundling transitions may play a role in conditions where keratin filament interactions are impaired, such as in epidermolysis bullosa simplex^50^. Beyond potential physiological implications, the physics underlying the star-bundling transition could inform the design of bioinspired polymeric materials. By harnessing the mechanisms identified in this study, such materials could be engineered to display transitions between states with markedly distinct material properties, along with controllable transition times and operating length scales.

## Methods

### Cell culture

MDCK strain II cells were used throughout the study. To visualize intermediate filaments, we used MDCK cells expressing keratin-18–GFP^29^. To visualize the plasma membrane, we used MDCK keratin-18–GFP co-expressing CAAX-iRFP. To test the effects of plectin, we used MDCK keratin-18–GFP cells co-expressing P1f-8–BFP. All MDCK cell lines were cultured in Dulbecco’s modified Eagle’s medium (Gibco) with high glucose and L-glutamine supplemented with 10% v/v fetal bovine serum (Gibco), 100 µg ml^−1^ penicillin and 100 µg ml^−1^ streptomycin. Cells were maintained at 37 °C in a humidified atmosphere with 5% CO_2_. Live imaging of nuclei was performed by incubating cells (12 h, 1/1,000 dilution) with SPY650-DNA (Spirochrome). MDCK CAAX-iRFP cells were obtained via transposon insertion of iRFP-CAAX. MDCK P1f-8–BFP cells were obtained via transposon insertion of P1f-8–BFP. The P1f-8–BFP cassette was generated by replacing the EGFP of the plasmid pGR240^38,51^ by the EBFP2 of the Addgene plasmid 55242, via the polymerase chain reaction and Gibson assembly. All cell lines tested negative for mycoplasma contamination.

### Fabrication of soft silicone gels

Soft silicone gels were prepared based on previously published protocols^52,53^. Briefly, silicone elastomer was synthesized by mixing a 1:1 weight ratio of CY52-276A and CY52-276B PDMS (Dow Corning Toray). After degassing for 30 min on ice, 120 µl of the gel was spin-coated onto glass-bottomed dishes (35 mm, no. 0 coverslip thickness, Mattek) for 10 s at 500 rpm and for 40 s at 5,000 rpm. The samples were cured at 65 °C overnight.

### Coating of soft PDMS gels with fluorescent beads

Previously cured soft PDMS gels were treated with (3-aminopropyl)triethoxysilane (APTES, Sigma-Aldrich) diluted at 5% in absolute ethanol for 3 min, rinsed three times with 96% ethanol and dried in an oven for 30 min at 60 °C. Fluorescent carboxylate-modified beads (FluoSpheres dark red, Invitrogen) were filtered (220 nm) and sonicated in sodium tetraborate (3.8 mg ml^−1^, Sigma-Aldrich), boric acid (5 mg ml^−1^, Sigma-Aldrich) and 1-ethyl-3-(3-dimethylaminoprpyl)carbodiimide (0.1 mg ml^−1^, Sigma-Aldrich), as described previously^53,54^. The bead solution was placed for 5 min on the gels and then rinsed three times with type-1 water. The beads were passivated by incubation with Tris-buffered saline (Sigma-Aldrich) for 20 min at room temperature, rinsed three times with type-1 water and dried in an oven for 15 min at 60 °C.

### Micropatterning of soft PDMS gels

Stamps for micropatterning dome footprints were fabricated from SU8-50 masters containing 100-µm-diameter cylinders that were raised by conventional photolithography. Uncured PDMS mixed at a 1:9 curing agent to elastomer weight ratio (Sylgard 184 Silicone Elastomer Kit, Dow Corning) was poured onto the masters and cured overnight at 65 °C. The cured PDMS was peeled off and cut into individual stamps. For the patterning, the PDMS stamps were incubated with 40 µg ml^−1^ fibronectin (from human plasma, Sigma-Aldrich) and 20 µg ml^−1^ fibrinogen Alexa Fluor 647 conjugate (Invitrogen) in phosphate-buffered saline (PBS; Sigma-Aldrich) for 1 h and then brought into contact with polyvinyl alcohol (Sigma-Aldrich) membranes to transfer the protein. The membranes treated with polyvinyl alcohol were gently placed on the soft PDMS gels for 1 h and dissolved in a 0.2% w/v solution of Pluronic F127 (Sigma-Aldrich) overnight to passivate the unpatterned surface. Afterwards, the gels were washed with PBS.

### Fabrication of a microfluidic pressure device

A detailed protocol for fabricating the microfluidic device that was to apply hydrostatic pressure onto cell monolayers has been described previously^55^. The device consists of five parts. From top to bottom, the first part is a thick PDMS block with four inlets and a channel connected to two inlets on its bottom. The second is a 100-µm-thick PDMS layer with a 5 × 5 square pattern of 80-µm-diameter holes and with two inlets passing through that layer. The third part is polycarbonate membrane with 400-nm-sized holes (Whatman Nuclepore Track-Etch Membrane 0.4 µm). The fourth part is a roughly 120-µm-thick PDMS layer with a channel perpendicular to the upper block channel, and the fifth part is a glass-bottomed dish (35 mm dish no. 0 glass, Cellvis).

The top block was made via replica moulding in a 3D-printed mould produced by vat polymerization with a digital light processing 3D printer (Solus DLP 3D Printer with SolusProto resin). The surface of the mould was silanized to prevent adhesion with unpolymerized PDMS. PDMS was mixed at a 1:9 curing agent to elastomer weight ratio and degassed for 30 min while the mould was preheated to 90 °C. The degassed PDMS was then poured into the mould, degassed again for 30 min and cured on a hotplate at 90 °C for at least 30 min. After curing, the PDMS was gently removed from the mould, cut into individual blocks and punched with a 1.5 mm Rapid-Core Sampling Tool (Electron Microscopy Sciences) to generate four inlets. The second PDMS part was fabricated from SU8-50 masters containing 80-µm-diameter columns. For one wafer, 10 ml elastomer was mixed with 1 ml curing agent, degassed for 30 min and distributed onto the wafer so that it covered all features. The wafer was then spun at 600 rpm for 1 min in a spin coater (WS-650MZ 23NPP/LITE, Laurell Tech), and an air gun was used to gently remove uncured PDMS from the top of all features. The wafer was then kept on a levelled hotplate overnight at room temperature before curing at 90 °C for 30 min the next morning. The top block and this part were connected by treating both pieces for 1 min with plasma in an ozone plasma cleaner (PCD-002-CE, Harrick Plasma). Afterwards, they were brought into direct contact with each other and bonded for 2 h at 80 °C.

The bottom layer with one channel was made by mixing PDMS in a 2:8 curing agent to elastomer ratio and pouring 3.5 ml onto a plastic dish (tissue culture dish 150, TPP), distributing and removing air bubbles with an air gun, keeping the plastic dish on a levelled surface overnight at room temperature, and curing for at least 1 h at 80 °C. After curing, the thin PDMS sheets were placed on a Silhouette cutting mat and cut into individual pieces on a Silhouette cutting machine (Silhouette Cameo 4, Silhouette America). The resulting pieces were connected to the glass-bottomed dish via plasma bonding for 1 min and resting in an oven for 2 h at 80 °C.

The polycarbonate membrane was cut into small pieces big enough to cover the intersection of two perpendicular channels and activated in a plasma cleaner for 1 min at 600 mTorr. They were then covered by a preheated 80 °C warm mixture of 5% APTES in Milli-Q water for 20 min before drying on clean-room tissue. These coated membranes were bonded to the top block by plasma-cleaning the block for 20 s at 600 mTorr and bringing the previously plasma treated side of the membrane in contact with the block. The remaining two pieces—the top block with the thin PDMS layer and the membrane plus the glass-bottomed dish with the bottom channel PDMS layer—were finally connected by plasma-cleaning both parts for 30 s and gently pressing them together without breaking the glass of the glass-bottomed dish. To increase stability, the base of the resulting device was surrounded by uncured PDMS and placed in an oven for 2 h at 80 °C.

### Patterning of the microfluidic pressure device

Surface protein patterns were applied inside the microfluidic devices via light-induced molecular adsorption of proteins with PRIMO (Alveole). The devices were first filled with 96% ethanol to remove bubbles and then washed with Milli-Q water. Afterwards, they were incubated with poly-L-lysine solution (Merck) for at least 1 h at room temperature. Next, they were washed with HEPES buffer (pH 8.4) and incubated with a freshly prepared solution of mPEG-succinimidyl valederate (Laysan Bio) in the same HEPES buffer at 50 mg ml^−1^ overnight at 4 °C. Directly before using PRIMO, the devices were washed with Milli-Q water and incubated with the photo-initiator (4-benzoylbenzyl)trimethylammonium chloride (Angene). The actual pattern was applied with PRIMO by illuminating the sample with an ultraviolet laser using the highest intensity on the area surrounding the dome foodprints and using 0% or 10% of the intensity on the foodprints. The ultraviolet-treated devices were then washed with PBS and incubated with an ice-cold solution of fibronectin (100 µg ml^−1^) and fibrinogen Alexa Fluor 647 (30 µg ml^−1^) in PBS for 5 min. After washing with PBS, the devices were used immediately for cell seeding or were stored overnight at 4 °C.

### Cell seeding

Samples were sterilized for 15 min under ultraviolet light and washed with sterile PBS directly before seeding. For spontaneous domes on soft PDMS, 80 µl of cells in medium at a concentration of 2,000,000 cells per millilitre were placed on the gel, incubated for 50 min, washed with PBS to remove unattached cells and incubated with 2 ml of Eagle’s minimal essential medium (Gibco) supplemented with 10% v/v fetal bovine serum (Gibco), 100 µg ml^−1^ penicillin and 100 µg ml^−1^ streptomycin.

For directed domes in microfluidic devices, 35 µl of cells in medium at a concentration of 30,000,000 cells per millilitre were added to the bottom channel and incubated for 50 min. Afterwards, the samples were washed with medium to remove unattached cells. If the seeding did not result in a confluent cell layer with the dome footprints being mostly unoccupied, the seeding was repeated once with the same parameters. The samples were then incubated for 24 h before the experiment.

### Directed dome experiments

One day before the experiment, a reservoir of CO_2_ independent medium (ThermoFisher) supplemented with 10% v/v fetal bovine serum (Gibco), 100 µg ml^−1^ penicillin, 100 µg ml^−1^ streptomycin and 1.3% v/v HEPES solution (Sigma-Aldrich) was incubated overnight at 37 °C and degassed for at least 30 min the next morning to remove all dissolved gas from the medium and prevent bubbles from forming in the tubes during the experiment. The cell medium in the microfluidic device was replaced by the same CO_2_ independent medium. After mounting the microfluidic chip on the stage of the temperature-controlled microscope, the reservoir of CO_2_ independent medium was connected with a silicone tube to the inlet of the pressure channel. A second silicon tube with a valve was then connected to the outlet of the pressure channel. A weak vacuum was connected to the outlet valve to remove all bubbles in the tubes, and the outlet valve was closed. Hydrostatic pressure was induced by increasing the hight of the reservoir relative to the height of the microfluidic device with the help of a vertically mounted motorized stage. The stage height for zero pressure was determined by increasing the height until domes formed and then gradually lowering the stage height until the domes disappeared, thus marking 0 Pa. The hydrostatic pressure during the experiments was determined with the conversion of 1 mm → 10 Pa based on Pascal’s law.

For latrunculin A experiments, 1 µM of latrunculin A (Sigma) dissolved in 200 µl of CO_2_ independent medium was added gently with a pipette directly into the cell channel.

### Immunostainings

Spontaneous domes were fixed with 4% paraformaldehyde in PBS for 10 min at room temperature and permeabilized with 0.1% Triton X100 (Sigma-Aldrich) in PBS for 10 min at room temperature. Afterwards a blocking solution of 1% bovine serum albumin (Sigma-Aldrich) in PBS was used for 1 h at room temperature. F-actin was labelled by adding tetramethylrhodamine phalloidin (Sigma-Aldrich) at 1:1,000 dilution in PBS for 30 min at room temperature. Nuclei were labelled by adding Hoechst (Hoechst 33342, Invitrogen) at 1:2,500 dilution in PBS for 10 min.

Imaging was performed on a confocal microscope (LSM880, Zeiss) running the software ZEISS2.3SP1FP3 (black, version 14.0.24.201) using the FastAiryScan mode and a plan-apochromate ×63 1.4-numerical-aperture oil immersion objective. Dome footprint positions were obtained with a 631-nm laser scan to visualize the fibrinogen Alexa Fluor 647 conjugate. Three channels were acquired: 405 nm to excite Hoechst, 488 nm to excite keratin-18–GFP and 561 nm to excite tetramethylrhodamine phalloidin. *Z*-stacks of all three channels were obtained sequentially with a step size of 144 nm.

### Time-lapse microscopy

These experiments were carried out on a confocal microscope (LSM880, Zeiss) running the software ZEISS2.3SP1FP3 (black, version 14.0.24.201) using the FastAiryScan mode. For the live directed domes, imaging was performed with a LD-LCI plan-apochromat ×40 1.2-numerical-aperture water immersion objective or a plan-apochromate ×63 1.4-numerical-aperture oil immersion objective. Dome footprint positions were obtained with a 561-nm laser scan to visualize the fibrinogen Alexa Fluor 546 conjugate. Depending on the experiment, the following channels were acquired: 488 nm to excite keratin-18–GFP, 631 nm to excite SPY-650 DNA, 631 nm to excite CAAX-iRFP and 405 nm to excite P1f-8–BFP. To prevent photodamage, the 405-nm channel was used only in the first and the last time points to provide a reference. *Z*-stacks were acquired with a 500-nm step size and different channels were acquired sequentially.

### Segmentation and tracking

Cell segmentation was performed with Cellpose^56^. For the fixed spontaneous domes, cells were segmented based on the maximum projection of the actin staining and the cyto1 model. For live directed domes, cells were segmented based on the maximum projection of the membrane marked and a cyto2-based model with human-in-the-loop retraining^57^. All resulting label maps were checked and, if necessary, corrected by hand. In the absence of a membrane or actin signal, cells were segmented by hand based on the keratin signal. Tracking was performed with the ImageJ plugin trackmate^58^ based on the label maps. All tracks were checked and, if necessary, corrected by hand.

### Image analysis

*R*_i_ and *θ* were measured for images of the keratin signal from individual cells after cropping based on the segmentation results (Extended Data Fig. 3). All analyses were performed in MATLAB 2020b (MathWorks), unless specified otherwise. The cell centre was determined as the centre of mass of the fluorescent intensity. From this centre, the image was transformed into polar coordinates, which generated a rectangular matrix such that each row has the same distance to the pole and each column has the same polar angle. To generate a perfect rectangle and avoid analysis artefacts at the cell edge, all columns were interpolated so that the cell edge reached the full length of the column. This essentially transformed the cell shape into a circle.

From the obtained rectangular matrix, we measured the mean intensity of each row to yield an intensity profile as a function of radial distance from the cell centre. We then rescaled the radius by its maximum and calculated *R*_i_ as the median of the resulting distribution. This resulted in a dimensionless value ranging from 0 to 1, where values near 1 indicate that keratin structures are predominantly localized at the cell periphery and values near 0 indicate central accumulation.

To obtain *θ*, the same rectangular matrix from the previous step was cropped so that it contained only the outer half. The resulting image was imported into ImageJ and smoothed with a Gaussian blur (*σ* = 4 px). We then measured the orientation of all keratin structures with the ImageJ plugin OrientationJ^59^ by calculating the vector field (tensor = 8, gradient = 0 and vectorgrid = 1), resulting in a local angle and coherency for every pixel^60^. *θ* was then calculated as the median of the absolute values of all local angles (as they are symmetric around 0°) weighted by their coherency. This yielded a single value between 0° (all keratin structures parallel to the cell edge) and 90° (all keratin structures perpendicular to the cell edge).

Both *R*_i_ and *θ* were used for the live data to differentiate between bundled and non-bundled cells. For each cell, the maximum average angle *θ*_max_ during the time series and the corresponding intensity radius *R*_corr_ at the same time point were measured. Cells were marked as being bundled if ${\theta }_{\max }$ exceeded a threshold ${\theta }_{{\rm{T}}}$ (set to 35° with slight adjustments between domes to avoid false positive or false negative detections) and ${R}_{\mathrm{corr}} < 0.45$ (see Supplementary Fig. 2 for an analysis of the sensitivity of bundling dynamics with ${\theta }_{{\rm{T}}}$). The same thresholds were then used to determine the bundling time *T*_B_, which was determined as the first time point at which both *R*_i_ and *θ* crossed their respective thresholds

The same analysis was performed for the 2.5D simulations by using the simulated images of the top view as input (Supplementary Note). For fixed data (Fig. 1), cells were classified as bundled, partly bundled or non-bundled via visual inspection.

To obtain the cell area in domes, the dome shape was measured with the ImageJ plugin Local Z Projector^61^, resulting in a height map. The label maps from the segmentation were converted into masks in ImageJ, and the actual cell area was calculated from the hight map and the cell masks with DeProj^61^ in MATLAB.

The offset of the nucleus referenced to keratin was obtained from cropped 3D stacks of individual cells containing both the keratin-18–GFP and the SPY650-DNA signal. The stacks were rotated in ImageJ, if necessary, so that the basal plane of the cell was parallel to the image plane. The central plane of the nucleus was then fitted with an ellipse, and the keratin signal was cropped to an area that had twice the lengths of the short and long axes of the fitted ellipse and with the same centre to avoid artefacts from cells with a curved basal plane. The sum of the signals from both channels of the rotated and cropped stacks was then measured as a function of the height in MATLAB, and the offset of the nucleus referenced to keratin intermediate filaments was calculated as the distance between the respective peaks. The final nuclear offset was then determined over time as a moving mean with window size 5.

### Calculating the tissue strain

Dome strain, defined as ${\varepsilon }_{{\rm{D}}}=\,{A}_{\mathrm{Dome}}/{A}_{\mathrm{Base}}-1$, was obtained by measuring the radius of curvature ${R}_{{\rm{C}}}$ and dome height H from the side view of the centre of the dome, where *A*_Dome_ = 2π*R*_C_ × *H* and *A*_Base_ = π(2*R*_C_ × *H*−*H*^2^). The dome strain was then calculated as ${\varepsilon }_{{\rm{D}}}=2{R}_{{\rm{C}}}/\left(2{R}_{{\rm{C}}}-H\right)-1$. The constitutive strain ${\varepsilon }_{{\rm{C}}}$ was obtained by measuring the mean cell area inside the dome footprint ${a}_{\mathrm{IN}}$ and the mean cell area outside the dome footprint ${a}_{\mathrm{OUT}}$ before inflation. The constitutive strain was then calculated via ${\varepsilon }_{{\rm{C}}}={a}_{\mathrm{IN}}/{a}_{\mathrm{OUT}}-1$. Finally, the tissue strain ${\varepsilon }_{{\rm{T}}}$ was calculated as the combination of both strains via ${\varepsilon }_{{\rm{T}}}=\,{\varepsilon }_{{\rm{D}}}+{\varepsilon }_{{\rm{C}}}+{\varepsilon }_{{\rm{D}}}{\varepsilon }_{{\rm{C}}}$. This calculation accounts for the fact that, before inflation, cells inside the footprint had a larger area than those outside it, as a consequence of sample preparation.

### Statistics

Comparisons between each group of unpaired samples were computed using the unpaired two-sided Wilcoxon rank sum test. Comparisons of multiple groups with each other were computed using the Kruskal–Wallis test. Paired samples were computed using the paired-sample *t*-test. Correlations were tested by computing Pearson’s correlation coefficient *R*. Statistical analyses were performed using MATLAB R2020b.

### Reporting summary

Further information on research design is available in the Nature Portfolio Reporting Summary linked to this article.

## Online content

Any methods, additional references, Nature Portfolio reporting summaries, source data, extended data, supplementary information, acknowledgements, peer review information; details of author contributions and competing interests; and statements of data and code availability are available at https://doi.org/10.1038/s41567-026-03371-8.

## Supplementary information

Supplementary Information Supplementary Figs. 1 and 2, Video captions 1–4 and Note.

Reporting Summary

Supplementary Video 1 Cell in a directed dome undergoing the bundle transition. Keratin-18–GFP signal with the result of the membrane segmentation in green.

Supplementary Video 2 Growth of a keratin-depleted zone. Keratin-18–GFP signal with the result of the membrane segmentation in green. The field of view is a zoom-in on one tricellular junction of the cell displayed in Supplementary Video 1.

Supplementary Video 3 Isometric view of a 3D model simulation. A single cell with the keratin fibres in green, the nucleus in cyan and the cell borders in grey. The cell is stretched gradually up to a maximum of 1,000%. During the stretch, the keratin fibres reorganize into a star-like bundle structure while the nucleus is pushed out of its keratin cage. A representative example of the pressure on the nucleus is plotted simultaneously against the strain.

Supplementary Video 4 Collective bundling process. Cropped images of keratin-18–GFP in a directed dome. The outlines indicate segmented cell borders colour-coded according to the average angle *θ*. The colour changes from blue for non-bundled cells to magenta for cells that crossed the bundling threshold.

## Source data

Source Data Fig. 1 Source data.

Source Data Fig. 2 Source data.

Source Data Fig. 3 Source data.

Source Data Fig. 4 Source data.

Source Data Extended Data Fig. 3 Source data.

Source Data Extended Data Fig. 4 Source data.

Source Data Extended Data Fig. 5 Source data.

Source Data Extended Data Fig. 6 Source data.

Source Data Extended Data Fig. 7 Source data.

Source Data Extended Data Fig. 10 Source data.

## Data Availability

The data that support the findings of this study are available from the corresponding authors on reasonable request and available via IBEC at https://doi.org/10.34810/DATA3386. Source data are provided with this paper.

## Code availability

Single-cell models are available via GitLab at https://gitlab.com/marco.pensalfini1/cytosim/-/tree/master/doc/papers/2025_Golde. All other analysis procedures and codes are available from the corresponding authors on reasonable request.
