# Supplementary material for: Dynamics of supracellular keratin bundling and nuclear uncaging in stretched epithelia
Source: Nat Phys. 2026 Jul 27;22(9):1436–45. doi: 10.1038/s41567-026-03371-8 (PMC13558057; doi:10.1038/s41567-026-03371-8)
Supplement: Supplementary file 1 [file 41567_2026_3371_MOESM1_ESM.pdf]

nature physics

Supplementary information https://doi.org/10.1038/s41567-026-03371-8

Dynamics of supracellular keratin bundling and nuclear uncaging in stretched epithelia

In the format provided by the authors and unedited

This file contains:

Supplementary figures 1-2

Supplementary video captions

Supplementary note

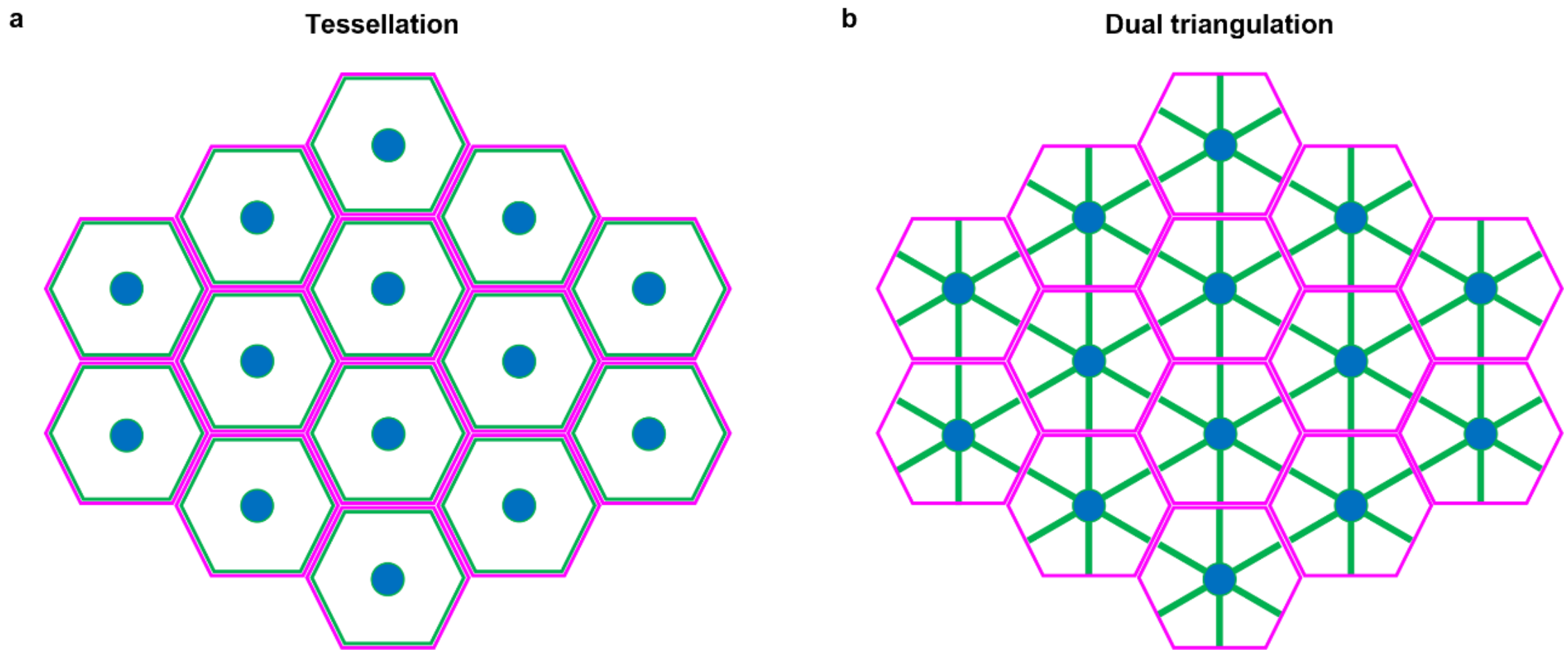

Supplementary Figure 1: Tessellation vs dual triangulation. a, In non-bundled cells, the keratin rim (green) forms a tessellation parallel to cell edges (magenta) with the nucleus (blue) as the approximate cell centre. **b**, In bundled cells, the keratin bundles (green) now form a dual triangulation that crosses the tessellation edges (magenta) and connects the central knots in the cell centre (blue). In this configuration, the bundles are the Delaunay triangulation of the centre points that corresponds to the dual graph of the Voronoi diagram represented by the cell edges.

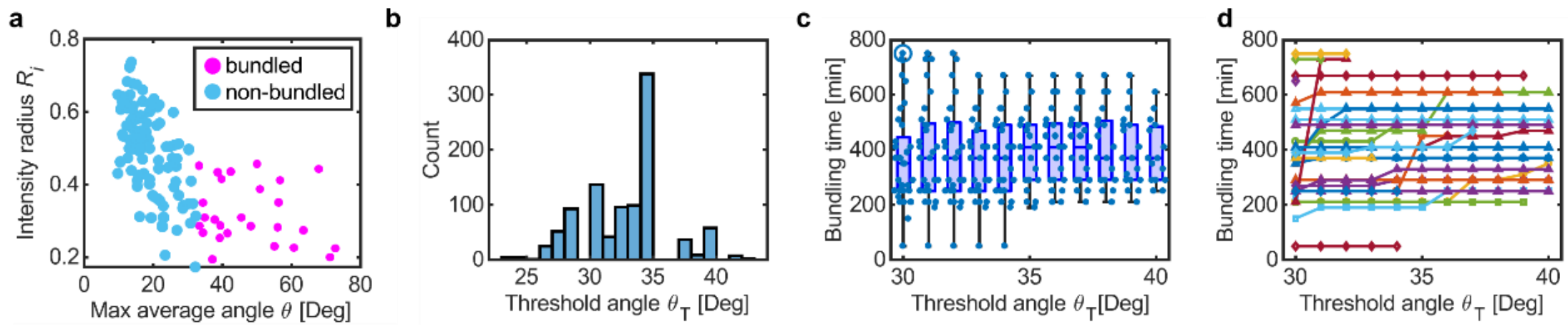

Supplementary Figure 2: Sensitivity of bundling dynamics to θ_T. a, To test the sensitivity of bundling dynamics with respect to the bundling threshold θ_T, we first identified, for each cell in the live dataset, the time point at which θ reached its maximum value. We then performed K-means clustering (k = 2), which yielded a threshold of $\theta_T = 33°$separating the two clusters. **b**, To assess the robustness of this threshold, we performed a bootstrap analysis in which θ_T was estimated from random subsets comprising 50% of the data. This yielded a distribution of thresholds with a peak between 34° and 35° and a standard deviation of 3.3°. **c**, Next, we examined how the bundling time varies when θ_T is changed within the range 30°- 40°. We found that the median bundling time remains stable across this range, indicating that the overall clustering dynamics do not depend sensitively on the specific choice of θ_T within a broad interval of threshold values. Significance was tested with Kruskal-Wallis test (all pairs are p>0.4). Boxes indicate the median and interquartile range, and whiskers extend to minimum and maximum values. **d**, Finally, we assessed whether our conclusion that clusters grow sequentially depends on the choice of threshold. To this end, we examined the bundling time of each cell across all domes as a function of θ_T. Here, individual cells are connected with a line of the same colour while individual domes are represented by different markers (circle, triangle, square, diamond). For the majority of cells, the measured bundling time is largely insensitive to the choice of θ_T, with only a small subset exhibiting an increase in bundling time at higher θ_T. Overall, this analysis indicates that the sequential nature of the clustering process is a robust feature of the data. n = 127 cells from 4 domes

Supplementary Videos

Supplementary Video 1: Cell in a directed dome undergoing the bundle transition. Keratin-18-GFP signal with the result of the membrane segmentation in green.

Supplementary Video 2: Growth of a keratin depleted zone. Keratin-18-GFP signal with the result of the membrane segmentation in green. The field of view is a zoom on one tricellular junction of the cell displayed in supplementary video 1.

Supplementary Video 3: Isometric view of a 3D model simulation. A single cell with the keratin fibres in green, the nucleus in cyan and the cell borders in grey. The cell is stretched gradually up to a maximum of 1000%. During the stretch, the keratin fibres reorganize into a star-like bundle structure while the nucleus is pushed out of its keratin cage. A representative example of the pressure on the nucleus is plotted simultaneously against the strain.

Supplementary Video 4: Collective bundling process. Cropped images of keratin-18-GFP in a directed dome. The outlines indicate segmented cell borders colour-coded according to the average angle θ. The colour changes from blue for non-bundled cells to magenta for cells that crossed the bundling threshold.

Supplementary Note

2.5D computational models

Generation of entangled networks

To establish 2.5D computational models of intermediate filament reorganization in the region around the medial cell plane, we leveraged a previously described model generation procedure to obtain 8 realizations of keratin networks with degree of entanglement $\mathcal{E} \approx 0.5$, as defined in reference [1]. Each model comprises 100 cylindrical and inextensible fibres interacting sterically through a harmonic potential of stiffness $k_s = 10$ nN/μm, each representing a keratin fibre (a collection of bundled filaments) of reference length $\ell_0 = 25$ μm, persistence length $\ell_p = 5$ μm, and diameter $\phi = 100$ nm. The base of the confining prism is a regular hexagon with reference apothem length $a_0 = 5$ μm and its height is $h_0 = 1.25$ μm. The nucleus is modelled as a cylindrical region of radius $r_n = 3.5$ μm which cannot be penetrated by keratin filaments, aligned with the prism height and centred with respect to the hexagonal base. The cytosolic space, corresponding to the cell region between the outer prism and the nucleus, is modelled as an environment of effective viscosity $\nu = 1$ pN s / μm^2 and all filament points are confined inside it by a harmonic potential whose stiffness is $k_c = 1$ nN/μm for points outside this space region and 0 for points located inside. Desmosomal junctions connecting intermediate filaments in neighbouring cells are modelled by ensuring that each fibre end remains attached to the cell lateral faces without being able to cross tricellular junctions. Following generation of the entangled networks, long-term equilibration ($t_{eq} = 100$ s) at room temperature ensures elimination of any pretension.

Formation of rim-and-spoke structures

To study the emergence of rim-and-spoke structures from initially random networks, we further allowed fibres to interact electrostatically over a distance $d_a = 100$ nm by activating an attractive potential of stiffness $k_a = 10$ pN/μm and modelled the presence of plectins on the nuclear surface and on the cell cortex. These were represented as uniformly distributed springs with a surface density of 100 μm^{-2} and stiffness $k_p = 10$ pN/μm. In our models, plectins may stochastically form bonds with any fibre point located within a distance d_b according to a binding rate $K^+ = 1$ s^{-1} and unbind with rate K^-. We then let the system equilibrate for $t_{eq} = 100$ s $\gg 1/K^+$ to track the self-organization of intermediate filaments under various choices for d_b (range: 0.25–1 μm) and K^- (range: 0.1–10 s^{-1}).

Stretching of 2.5D cell models

In the steps described above, the axial tension in fibres and the forces on desmosomes are relatively small. Because this may not be the case during cell

stretching, we enabled fibre extensibility with axial stiffness $k = 1$ nN/μm, and increased the stiffness of the confinement potential by one order of magnitude ($k_c{}' = 10$ nN/μm) to preserve the larger stiffness of desmosomal junction with respect to intermediate filaments. We also modelled plectin bonds as slip bonds, with a force-dependent unbinding rate $K_{eff}^{-} = K^{-} \exp(f/f_{ub})$, where $f_{ub} = 100$ pN is a force-sensitivity parameter. Then, we rapidly increased the cell base area by 4-fold over a time span of 6 s, after which the stretched configuration was maintained for 25 s. The cell height was kept constant throughout these simulations.

3D computational models

Generation of entangled networks

To better investigate the interaction between keratin filaments and the nucleus, we obtained 7 realizations of fully 3D computational models comprising entangled networks ($\mathcal{E} \approx 0.5$) of 200 keratin fibres with reference length $\ell_0^{3\mathrm{D}} = 35$ μm. The nucleus is represented as a rigid sphere of radius $r_n = 3.5$ μm interacting sterically with the fibres and the cell space is a hexagonal prism of reference height $h_0^{3\mathrm{D}} = 10$ μm. All other physical parameters have the values already indicated for the generation of 2.5D models and a long-term equilibration ($t_{eq} = 100$ s) phase at room temperature is also applied to ensure elimination of any pretension within the fibre network.

Formation of rim-and-spoke structures and apical-basal asymmetry

Contrary to the 2.5D models and motivated by the need to maintain a manageable computational cost for the 3D simulations, we represented the interaction of the fibres with the cell cortex and the nucleus by introducing a short-ranged attractive potential on both surfaces. This attracts any fibre point located inside the cell but outside the nucleus that is within a distance $d_b^{3\mathrm{D}} = 0.5$ μm from the nuclear surface with a stiffness $k_c^{3\mathrm{D}} = 10$ pN/μm. Consistently with the 2.5D simulations, we also let these models equilibrate for $t_{eq} = 100$ s to give keratin fibres time to self-organize into rim-and-spoke structures.

Then, we induced the apical-basal asymmetry that is typically observed in epithelial cells by deactivating the attractive potential acting on the top surface of the cell space enclosing the intermediate filament network and gradually halving the height of the lateral cortex over a time period of 10 s. The obtained model was then equilibrated for $t_{eq} = 100$ s.

Stretching of 3D cell models

To simulate cell loading, we enabled fibre extensibility ($k^{3\mathrm{D}} = 10$ nN/μm), increased the stiffness of the confinement potential by one order of magnitude ($k_c{}' = 10$ nN/μm), and we gradually increased the cell area to reach 1000% strain in 10 s.

Computational model visualization as image projections

To quantitatively analyse our computational predictions of keratin spatial distribution and compare them with experimental measurements, we used our customized version of Cytosim to extract 75-nm-thick slices of the model at each simulation time step. By treating the intermediate filament, cell cortex, and nucleus signal channels separately, we first obtained image stacks along the top-bottom (2.5D and 3D models) and front-back (3D model only) directions and then combined using Fiji's image stack projection utility [2]. "Sum slices" was used for each channel in the 2.5D models, while a maximum intensity projection was preferred for the nucleus signal in the 3D models. Regardless of the chosen method, the obtained projections were merged into composite images. These were used for model visualization and, in the case of 2.5D simulations, served as input for further quantitative analysis using the Matlab algorithm described in the "Image analysis" section.

Monte-Carlo-simulations

Monte-Carlo-simulations were performed in Matlab R2020b. We first generated a hexagonal grid with 19 cells and a next neighbour list based on shared interfaces. The bundling times of 4 different domes as presented in Fig. 4b were used as input parameters. Each dome was simulated 1000 times, resulting in a total of 4000 simulations. At the start of the simulation, all cells were in a non-bundled state. Using the bundling times in sequence, non-bundled cells were selected to switch into a bundled state until all bundling times of the dome were used. In the unbiased scenario, this selection was performed randomly with an equal chance for every non-bundled cell to be selected. Consequently, bundled cells could not be selected again and stayed in the bundled state. During the simulation, the time and position of each bundled cell was logged. After the simulation, the number of nucleation events of each dome was determined by counting how many cells bundled without having previously bundled neighbours with the help of the next neighbour list.

In the biased scenario, cells were divided into two populations: cells being neighbours of already bundled cells and free cells without a connection to already bundled cells. These populations were updated after every bundling event with the help of the next neighbour list. A conditional probability to select cells from the neighbour population was used as an input parameter with values between 0 and 1. During the simulation, a random number between 0 and 1 was generated for every bundling event. If this number was below the conditional probability, a random cell from the neighbour pool was selected. If the number was above the conditional

probability, a random cell from the free pool was selected. If one of the pools had no elements left, a random cell from the other pool was selected. The number of nucleation events was determined in the same way as in the unbiased scenario.

Supplementary References

1. Pensalfini, M., Golde, T., Trepat, X. & Arroyo, M. Nonaffine Mechanics of Entangled Networks Inspired by Intermediate Filaments. *Phys. Rev. Lett.* **131**, 058101 (2023).
2. Schindelin, J. *et al.* Fiji: an open-source platform for biological-image analysis. *Nat. Methods* **9**, 676–682 (2012).
